# A specific phosphorylation-dependent conformational switch in SARS-CoV-2 nucleocapsid protein inhibits RNA binding

**DOI:** 10.1126/sciadv.aax2323

**Published:** 2024-08-02

**Authors:** Maiia Botova, Aldo R. Camacho-Zarco, Jacqueline Tognetti, Luiza Mamigonian Bessa, Serafima Guseva, Emmi Mikkola, Nicola Salvi, Damien Maurin, Torsten Herrmann, Martin Blackledge

**Affiliations:** Université Grenoble Alpes, CNRS, CEA, IBS, F-38000 Grenoble, France.

## Abstract

The nucleocapsid protein of severe acute respiratory syndrome coronavirus 2 encapsidates the viral genome and is essential for viral function. The central disordered domain comprises a serine-arginine–rich (SR) region that is hyperphosphorylated in infected cells. This modification regulates function, although mechanistic details remain unknown. We use nuclear magnetic resonance to follow structural changes occurring during hyperphosphorylation by serine arginine protein kinase 1, glycogen synthase kinase 3, and casein kinase 1, that abolishes interaction with RNA. When eight approximately uniformly distributed sites have been phosphorylated, the SR domain binds the same interface as single-stranded RNA, resulting in complete inhibition of RNA binding. Phosphorylation by protein kinase A does not prevent RNA binding, indicating that the pattern resulting from physiologically relevant kinases is specific for inhibition. Long-range contacts between the RNA binding, linker, and dimerization domains are abrogated, phenomena possibly related to genome packaging and unpackaging. This study provides insight into the recruitment of specific host kinases to regulate viral function.

## Teaser

Phosphorylation of SARS-CoV-2 nucleocapsid protein induces conformational changes that inhibit RNA binding and release bound RNA.

## INTRODUCTION

Severe acute respiratory syndrome coronavirus 2 (SARS-CoV-2), the infectious agent responsible for the recent COVID-19 pandemic, is an enveloped positive-sense single-stranded RNA virus of the betacoronavirus genus that expresses its own replication machinery. Genome replication is achieved by the RNA-dependent RNA polymerase, whose structure has been investigated by cryo–electron microscopy (*1*–*3*), within viral replication organelles called double-membrane vesicles (DMVs) that are remodeled from the endoplasmic reticulum (*4*). DMVs have been shown to constitute principal sites of viral RNA replication (*5*, *6*), containing narrow exit channels formed by nonstructural viral proteins (nsps) through which newly synthesized single-stranded RNA can exit before encapsidation.

The nucleocapsid protein is the most abundant protein in beta-coronaviruses and an important cofactor of the replication machinery (*7*, *8*) that is known to colocalize to the replication transcription complex (*9*–*11*) via an essential interaction with the N terminus of nonstructural protein 3 (nsp3) (*12*, *13*). SARS-CoV-2 nucleocapsid protein (N) binds to, and encapsidates, the viral genome, shielding the RNA from the host innate immune system, as well as playing an essential but still poorly understood role in regulation of gene transcription (*14*). Electron tomography was used to identify N-RNA complexes within SARS-CoV-2 virions, displaying a morphology resembling beads on a string (*15*, *16*).

**Fig. 1.**
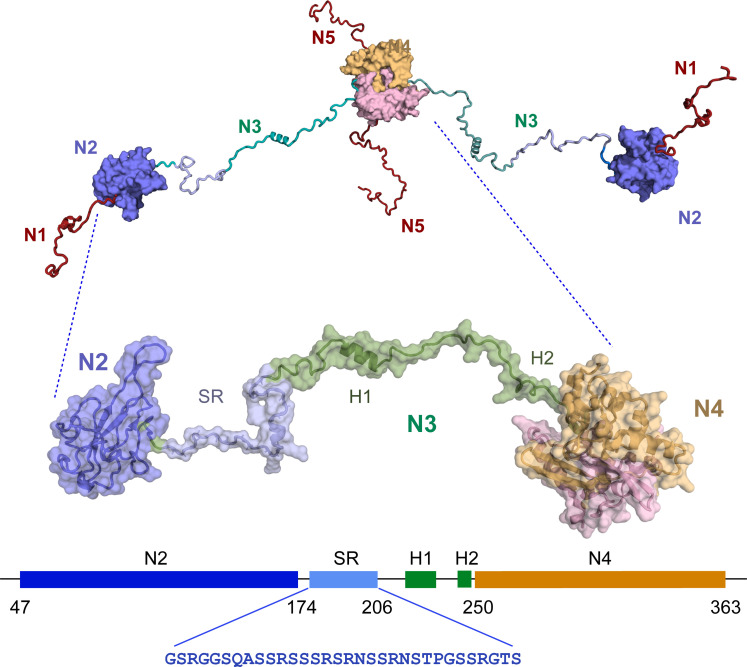
Figurative representation of SARS-CoV-2 nucleocapsid protein (N). SARS-CoV-2 nucleocapsid protein comprises five domains (N1 to N5, top). The construct shown in the zoom comprises three of these domains, N2, the RNA binding domain; N3, the disordered central domain containing two helices H1 (219 to 231) and H2 (248 to 255) and the serine-arginine–rich (SR) region that is hyperphosphorylated in infected cells; and the dimerization domain N4. This construct is referred to as N234. The sequence of the SR region is shown in blue.

N is highly disordered as revealed by nuclear magnetic resonance (NMR) spectroscopy (*17*, *18*), comprising three intrinsically disordered domains (N1, N3, and N5) (Fig. 1), flanking the RNA binding domain (N2) and the dimerization domain N4, whose structures have both been determined at atomic resolution (*19*–*23*). RNA binding to N2 has been described (*24*–*32*), including its potential role in liquid-liquid phase separation (LLPS) of N-RNA mixtures (*26*, *27*, *33*–*39*). N4 forms a highly stable dimeric structure involving two domain-swapped β hairpins (*22*, *40*). Although N2 is known to represent the main RNA binding domain, secondary RNA binding sites have been proposed to exist in both N3 (*26*, *37*, *41*, *42*) and N4 (*40*, *41*, *43*–*46*), an observation supported by the assembly of truncation mutants of N into large supramolecular complexes of the dimensions of ribonucleoproteins (RNPs) (*47*). N5 has also been associated with assembly into higher-order oligomeric states (*48*).

N3 is of particular interest for a number of reasons. It comprises three distinct linear segments, a serine-arginine–rich region (SR, S176 to S206), a hydrophobic, leucine-rich helix (H1), and a polar region, comprising a glutamine-rich strand and terminated by a lysine-rich region that forms a detached helix (H2) in the crystal structure of N4, and represents the junction between these two domains. N3 was recently shown to fold around the N-terminal, ubiquitin-like domain of nsp3 (Ubl1) (*49*). H1 binds tightly to a hydrophobic cleft on the surface of Ubl1, while the polyQ and H2 regions complete a bipartite folding-upon-binding interaction. The interaction results in a massive collapse in the conformational sampling of dimeric N, bringing N2 and N4 in close contact and forming a compact complex with Ubl1. Nsp3 forms the cytosolic extremity of the exit channel of DMVs, suggesting that this essential interaction between viral proteins plays a role in positioning N before encapsidation of the nascent RNA, a model supported by the observed accumulation of N in the vicinity of the surface of DMVs (*4*).

The functional importance of N3 has also been highlighted by the accumulation of important mutations, associated with dominant variants of concern in this region (*50*, *51*) (for example, alpha, gamma, omicron ^203^RG-KR, beta ^205^T-I, delta ^203^R-M, omicron ^215^G-C, and BA.2.86 ^229^Q-K). Most of these mutations are associated with the SR segment of N3. Indeed mini-replicon studies have measured 10-fold increased mRNA delivery and protein expression for commonly found mutants, and a reverse genetics model revealed 50-fold more virus production for S202R and R203M mutations (*52*). Helix H1 has also been linked to assembly, with a recent study using analytical ultra-centrifugation and circular dichroism combined with molecular dynamics simulation, suggesting that the motif promotes higher-order assembly (*53*).

Perhaps most importantly, the SR region of N3 is found to be hyperphosphorylated in infected cells, although it is unphosphorylated during viral assembly and is found in the unphosphorylated form when bound to genomic RNA in infectious virions (*54*–*56*). It has been suggested that hyperphosphorylation or dephosphorylation may play a role as a functional switch in the replication cycle of the virus, either between transcription and replication, or to enable genome packaging or unpackaging (*14*, *57*–*60*). Phosphorylation has also been shown to affect the compactness of viral ribonucleoprotein (vRNP) complexes (*61*), and concurrent studies on this have shown that binding of the isolated N3 to long RNAs is modulated by phosphorylation (*62*), while fluorescence polarization measured nearly an order of magnitude weaker binding to a 10-nucleotide (nt) RNA following hyperphosphorylation (*47*).

N is also known to undergo LLPS upon mixing with RNA (*26*, *27*, *33*–*38*), possibly providing compartmentalization of some stage of the viral replication cycle, as has recently been shown for negative-sense single-stranded RNA viruses, such as rabies (*63*) and measles (*64*). Hyperphosphorylation of the SR region (pSR) has also been shown to modulate the nature of membraneless organelles formed from N and RNA (*27*, *35*, *37*, *38*). Although the specific function of SARS-CoV-2 viral compartments remains the subject of debate (*65*), it is clear that the storage of high concentrations of N in the vicinity of DMVs (*4*, *6*, *16*, *55*) may provide an efficient mechanism for rapid encapsidation of newly synthesized RNA.

Despite the extensive work described above, little is known about the influence of phosphorylation on the structural and dynamic behavior of N and its impact on molecular function. The kinase cascade responsible for hyperphosphorylation of N was identified (*60*), implicating, sequentially, serine arginine protein kinase 1 (SRPK1), glycogen synthase kinase 3 (GSK-3), and casein kinase 1 (CK1). Here, we use time-dependent NMR spectroscopy, to characterize the conformational and functional implications of hyperphosphorylation of N at atomic resolution. This reveals that transient contacts between H1 and N2, and between N4 and N2 are abolished upon hyperphosphorylation and replaced by direct binding of pSR to the RNA binding surface of N2. pSR appears to interact with N2 via the same interface as single-stranded RNA, suggesting an auto-inhibitory mechanism involving direct competition with RNA. Notably, we demonstrate that phosphorylation by SRPK1 and GSK-3 is sufficient for this inhibition, although not all phosphorylation sites are required and that in vitro phosphorylation by noncognate kinases such as protein kinase A (PKA), a commonly used proxy for hyperphosphorylation, does not result in inhibition of RNA binding. In particular, time-resolved NMR reveals the precise threshold of the specific phosphorylation pattern necessary to achieve the competitive interaction mechanism required to fully inhibit RNA binding, providing important and unique insight into the role of posttranslational modification in the SARS-CoV-2 replication cycle.

## RESULTS

### The disordered SR region of N234 is hyperphosphorylated by SRPK1, GSK-3, and CK1

**Fig. 2.**
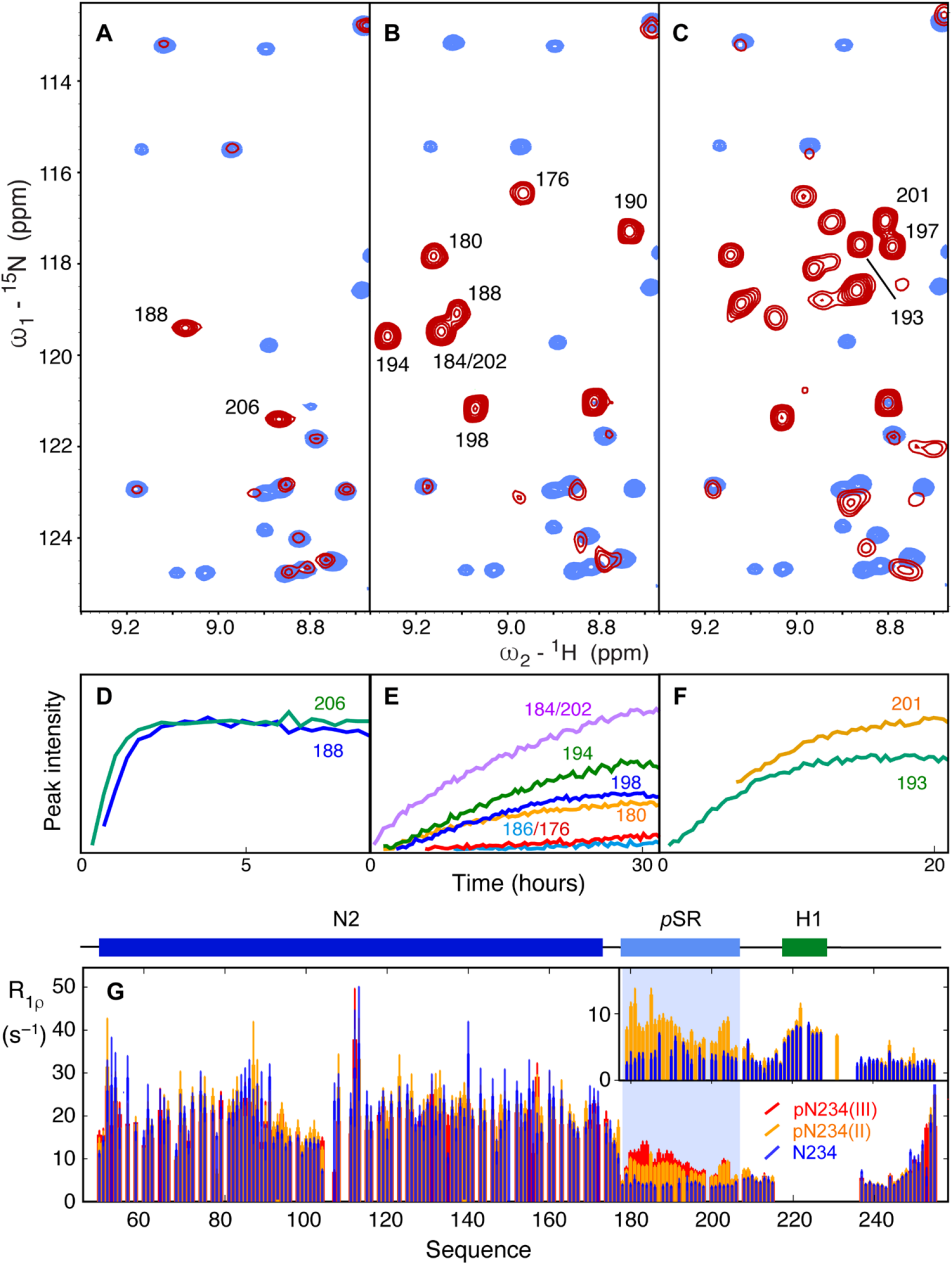
Phosphorylation of N by SRPK1, GSK-3, and CK1. (**A**) Comparison of ^15^N-^1^H HSQC of unphosphorylated (blue) and phosphorylated (red) N234 following incubation with SRPK1 and phosphorylation buffer [referred to as pN234(I)]. Two resonances appear that have been assigned to pS188 and pS206. (**B**) Comparison of ^15^N-^1^H HSQC of unphosphorylated (blue) and phosphorylated (red) N234 following incubation of pN234(I) with GSK-3 and phosphorylation buffer [referred to as pN234(II)]. Eight additional resonances appear (seven are shown here) that have been assigned to pS176, pS180, pS184, pS186, pS190, pS194, pT198, and pS202. (**C**) Comparison of ^15^N-^1^H HSQC of unphosphorylated (blue) and phosphorylated (red) N234 following incubation of pN234(II) with CK1 and phosphorylation buffer [referred to as pN234(III)]. Four additional resonances appear (three are shown) that have been assigned to pS193, pS197, and pS201. All spectra were buffer exchanged into standard NMR buffer before recording these spectra. (**D** to **F**) Evolution of peak intensity as a function of time for selected resonances that remain sufficiently resolved throughout the kinetic series to accurately measure peak intensities in each step (in phosphorylation buffer). (D) pN234(I), (E) pN234(II), and (F) pN234(III). (**G**) Phosphorylation modifies the dynamic behavior of N. Main panel: Rotating frame relaxation (R_1ρ_) of N234 at 850 MHz as a function of the phosphorylation state [blue, nonphosphorylated; orange, pN234(II); red, pN234(III)]. Data from N4 are not shown due to low signal-to-noise ratio. Inset: Rotating frame relaxation (R_1ρ_) of N123 at 950 MHz [blue, nonphosphorylated N123; orange, pN123(II)]. Helix H1 is visible in N123, probably due to the lower molecular weight of this construct, allowing comparison of the dynamic behavior of this region of N3. This comparison reveals that hyperphosphorylation strongly impacts the dynamic properties of the SR region.

A construct comprising domains N2, N3, and N4 (N234), including the RNA binding, dimerization, and hyperphosphorylation domains, was initially investigated (residues 45 to 375, lacking only the disordered N- and C-terminal domains). Incubation with SRPK1 resulted in the appearance of two phosphorylation sites that were assigned to sites pS188 and pS206. Published studies have demonstrated the sequential dependence of the three kinases, identifying that SRPK1 phosphorylates two sites that prime for subsequent phosphorylation of eight sites by GSK-3, which, in turn, primes for four more sites that are phosphorylated by CK1 (*60*). The addition of GSK-3 and then CK1 results in phosphorylation of eight and four additional sites, respectively, in agreement with recent reports (*59*) (Fig. 2). Under physiological conditions, the total theoretical charge of the 31–amino acid SR region thus evolves from +6, to +2, −14, and lastly −22 over the three phosphorylation steps [resulting in pN234(I), pN234(II), and pN234(III), corresponding to phosphorylation by SRPK1, GSK-3, and then CK1, respectively]. Each phosphorylation site was assigned using triple-resonance three-dimensional (3D) NMR, and real-time NMR was used to compare the rate of phosphorylation of different sites as a function of each kinase. In particular, this allows us to identify S176 and S186 that are phosphorylated notably slower by GSK-3 than S180, S184, S190, S194, S198, and S202 (Fig. 2E). Note that while the ^15^N-^1^H correlation peak of pS176 is resolved, and can therefore be accurately analyzed in terms of intensity buildup, pS186 is partially overlapped with pS184 and pS202 and notably slower than both, complicating further analysis (the nonphosphorylated peak is also strongly overlapped).

To investigate possible changes in backbone conformation, ^13^C^α^ chemical shifts of pSR were compared to recently proposed random coil chemical shift values (*66*, *67*) for unphosphorylated, pN234(II)-, pN234(III)-, and PKA-phosphorylated N234 (fig. S1), indicating that no obvious induction of secondary structure results from hyperphosphorylation, as has been previously observed for PKA-phosphorylated N (residues 1 to 209) (*46*).

### Hyperphosphorylation modifies the dynamic behavior of the disordered SR region

To investigate the impact of phosphorylation on the dynamic behavior of N234, we have measured ^15^N spin relaxation as a function of phosphorylation state (Fig. 2G). R_1ρ_ reports on local reorientational correlation times of internuclear ^15^N-^1^H bonds distributed along the primary sequence. This reveals that although N2 appears to maintain its hydrodynamic behavior, with comparable R_1ρ_ in the presence and absence of phosphorylation, the overall correlation time of the SR region increases over the three steps. Similar observations have been made in phosphorylation studies (*68*, *69*). The increase in pSR is more or less systematic for both pN234(II) and pN234(III), with local peaks observed around residues pS180, pS188, and 202 to 204. Comparison of R_1ρ_ measured from pN123(II) confirms the above observations and demonstrates that relaxation of the helical region H1 is not measurably affected by phosphorylation (resonances from this helix are too broadened in N234 to measure relaxation with sufficient precision).

### NMR maps the interaction of N234 with RNA from the viral 5′ UTR

**Fig. 3.**
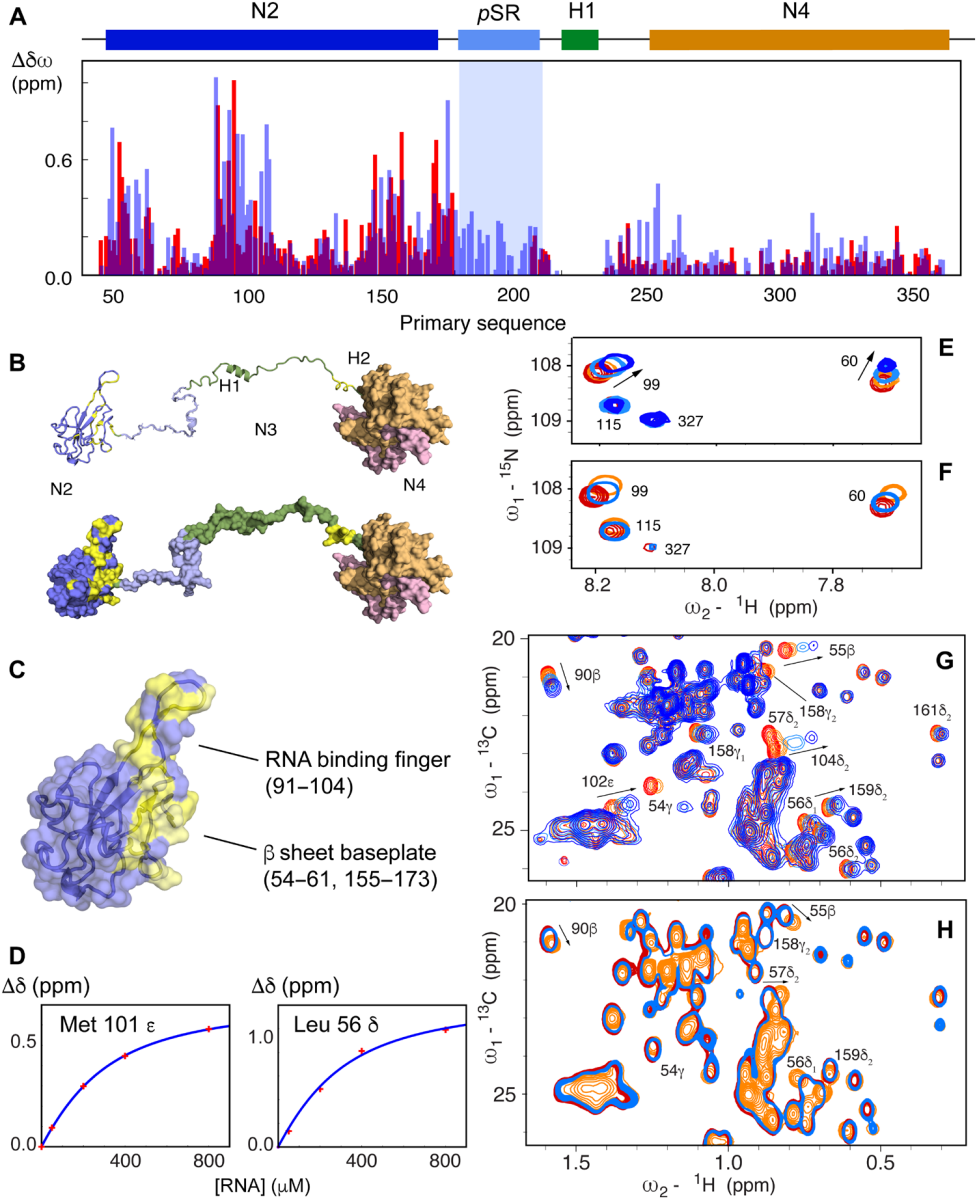
Hyperphosphorylation mimics RNA binding to N2. (**A**) CSPs measured in the ^15^N-^1^H TROSY spectrum of N234 following addition of single-stranded 14-mer RNA (UCUAAACGAACUUU) (blue) in comparison with chemical shift differences between nonphosphorylated and SRPK1/GSK-3/CK1 phosphorylated N234 (red) (the extreme shifts from the pSR region due to direct phosphorylation of the residue of interest are not shown for reasons of dynamic range). CSPs due to RNA binding were scaled by a factor 1.5 for optimal comparison with phosphorylation CSPs. (**B**) Mapping of the largest chemical shift differences onto N2 and N3 (yellow), shown in ribbon and surface representation. (**C**) Location of the RNA binding finger and baseplate β sheet on the RNA binding domain, N2. Yellow surface as in (B). (**D**) Examples of chemical shift titrations derived from ^13^C-^1^H HMQC of 150 μM N234 in the presence of increasing concentrations of 14-mer RNA. Residues in the RNA binding finger (Met^101^) and baseplate β sheet (Leu^56^) on the RNA binding domain are shown. The association constant derived from these titrations is (230 ± 10) μM. (**E**) CSPs in ^15^N-^1^H TROSY spectra of N234 (concentration, 150 μM) as a function of 14-mer single-stranded RNA concentration (red, 0%; orange, 25%; light blue, 100%; dark blue, 200%) (see fig. S8 for full spectra). (**F**) CSPs in ^15^N-^1^H TROSY spectra of N234 (concentration, 150 μM) for the same residues as (E) as a function of phosphorylation state [red, unphosphorylated N234; light blue, pN234(I); orange, pN234(II)]. (**G**) CSPs in ^13^C-^1^H HMQC spectra of N234 (concentration, 150 μM) for selected residues as a function of 14-mer single-stranded RNA concentration (red, 0%; orange, 25%; light blue, 100%; dark blue, 200%). (**H**) CSPs in ^13^C-^1^H HMQC spectra of N234 (concentration, 150 μM) for the same residues as (E) as a function of phosphorylation state [red, unphosphorylated N234; light blue, pN234(I); orange, pN234(II)].

We have investigated the RNA binding profile of N234 using a 14-nt segment (UCUAAACGAACUUU) from the 5′ UTR of SARS-CoV-2 and a 30-nt polyA. ^15^N-^1^H chemical shift perturbations (CSPs) in N2 measured in N234 (Fig. 3A) resemble those recently published by numerous authors (*25*, *28*–*31*, *45*), involving the RNA binding finger (residues 90 to 106), and the solvent-accessible surface of the baseplate formed by residues 48 to 66, 148 to 160, and 166 to 174. Additional shifts are seen throughout the SR region as well as in the boundary between N3 and N4 (residues 244 to 257), a lysine-rich region that folds as an α helix (H2) upon binding nsp3, and forms a detached α helix in the crystal structure of N4 (*20*, *21*). This region was also shown to interact with RNA in the context of isolated N4 (*44*), as well as in SARS-CoV (*41*). Apart from H2, CSPs in N4 are small, except for the solvent-exposed C-terminal helix comprising residues 345 to 357.

The binding interface is confirmed by ^13^C-^1^H methyl CSPs (Fig. 3G). Chemical shift titration of six methyl group signals as a function of concentration of added RNA reveals an affinity of (230 ± 10) μM (Fig. 3D). Titration with the two different RNAs resulted in shifts of the same amino acids in N2 and appears upon the same linear CSP trajectories (fig. S2); the exact position is dependent on the titration admixture and exchange regime that differs between RNAs ranging from fast to slow exchange on the NMR time scale, likely related to different affinities (the longer RNA was found to be in slower exchange).

### Hyperphosphorylated SR inhibits N:RNA interaction via the RNA binding surface of N2

A comparison of the ^15^N-^1^H and ^13^C-^1^H CSPs due to binding of diverse RNAs, and those resulting from sequential phosphorylation by SRPK1, GSK-3, and CK1 reveals remarkable correspondences (Fig. 3A). While the SRPK1 priming step induces small shifts (see fig. S3), the addition of 12 phosphorylation sites in pN234(III) results in a very similar distribution of CSPs in N2 compared to those observed as a result of RNA binding [chemical shifts in pN234(II) and pN234(III) show a similar profile, fig. S4]. This indicates that hyperphosphorylation induces a change in conformation of N2, probably due to binding of N2 to the pSR region, that mirrors the impact of RNA binding. We note that although the CSPs are distributed similarly, they can be different in sign to those observed upon binding RNA, demonstrating that while the same sites on the surface of N2 are affected, the bound state chemical shifts (RNA-bound and pN3-bound) are different (Fig. 3, G and H).

**Fig. 4.**
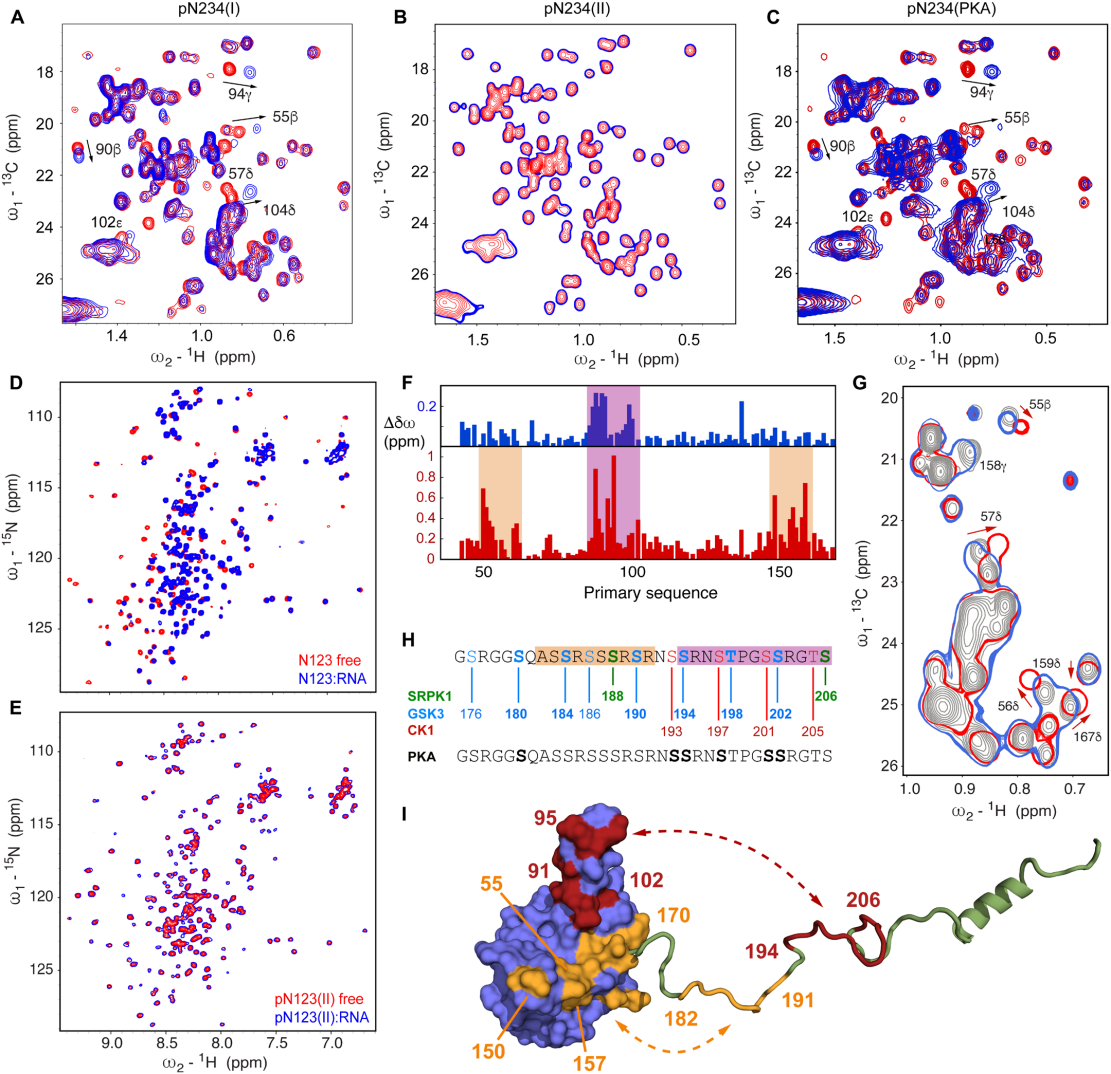
Hyperphosphorylation by SRPK1, GSK-3, and CK1 inhibits RNA binding. (**A**) ^13^C-^1^H HMQC spectra of N234 (concentration, 150 μM) for increasing concentrations of 14-mer single-stranded RNA (red, 0%; dark blue, 200%). (**B**) Chemical shifts in ^13^C-^1^H HMQC spectra of SRPK1/GSK-3 phosphorylated N234 [150 μM pN234(II)], for selected residues as a function of 14-mer single-stranded RNA concentration (red, 0%; dark blue, 200%). No notable shifts were observed [compare to (A)]. The 200% spectrum is presented as a single contour to highlight the lack of binding. (**C**) Chemical shifts in ^13^C-^1^H HMQC spectra of N234 phosphorylated by PKA (150 μM), for selected residues as a function of 14-mer single-stranded RNA concentration (red, 0%; dark blue, 200%). Shifts are highly comparable with CSPs measured in unphosphorylated N234 [compare to (A)]. (**D**) ^15^N-^1^H TROSY spectra of nonphosphorylated N123 (concentration, 200 μM) in the presence and absence of equimolar 14-mer single-stranded RNA (red, 0%; blue, 100%). (**E**) ^15^N-^1^H TROSY spectra of SRPK1-GSK3 phosphorylated N123 (concentration, 200 μM) in the presence and absence of equimolar 14-mer single-stranded RNA (red, 0%; blue, 100%). The 100% spectrum is presented as a single contour to highlight the lack of binding. (**F**) CSPs (blue) in ^15^N-^1^H TROSY of N234 phosphorylated by PKA, and (red) pN234(II). (**G**) Section of the ^13^C-^1^H HMQC spectrum of unphosphorylated N234 (gray), with N234 phosphorylated by PKA (blue) and pN234(II) (red). (**H**) Sequence of SR region showing in vitro phosphorylation sites (bold) following incubation with PKA (gray/black) and sequentially SRPK1 (green), GSK-3 (blue), and CK1 (red). (**I**) Sketch illustrating putative differential binding sites of N-terminal (182 to 191) and C-terminal (194 to 206) regions of pSR on the surface of N2. Red shading on N2 indicates CSPs induced by both PKA and SRPK1/GSK-3 phosphorylation, while orange indicates regions that are only shifted by SRPK1/GSK-3 phosphorylation.

We then tested the RNA binding properties of the different forms of pN234. While phosphorylation due to SRPK1 has no observable effect on RNA binding (Fig. 4A), the additional phosphorylation sites resulting from incubation with GSK-3 and CK1 result in complete abrogation of RNA binding (Fig. 4B). This was found to be the case for both RNA molecules that were tested. Both pN234(II) and pN234(III) fail to bind RNA, demonstrating that phosphZZorylation by SRPK1/GSK-3 is necessary and sufficient to inhibit RNA binding. We note that it has been previously observed that phosphorylation by SRPK1 is an essential step for phosphorylation by GSK-3 (*60*).

The broadly phosphorylating PKA has been exploited to phosphorylate N in a single step, including via coexpression with N (*46*), and notably to investigate the impact on binding of the host factor 14-3-3 (*70*). Incubation of N with PKA in vitro results in phosphorylation of six sites in the SR region of N234 (fig. S5) whose backbone resonances were assigned, namely, pS180, pS193, pS194, pS197, pS201, and pS202, although broader phosphorylation was observed by coexpression with PKA (*46*, *70*). This sixfold phosphorylated form of N234 nevertheless maintains its binding to RNA (Fig. 4C), with essentially identical spectral changes compared to the nonphosphorylated form in interaction with the same RNA (Figs. 3G and 4C and fig. S6). The specific pattern resulting from SRPK1/GSK-3 phosphorylation is therefore critical for abrogating RNA binding to N234, while N234 phosphorylated by PKA in vitro is unable to reproduce this functional phenotype.

### N2 and N3 are the minimum necessary elements for RNA binding inhibition

A construct comprising domains N1, N2, and N3 (N123, residues 1 to 267), including the N-terminal intrinsically disordered region, RNA binding, and hyperphosphorylation domains, was also investigated. The same signature of RNA binding is observed in N2 in N123 as in N234 (fig. S7), demonstrating that any impact of the dimerization domain, N4, or the disordered N-terminal domain, N1, on binding of these RNA segments is too weak to be observed by ^13^C-^1^H and ^15^N-^1^H CSPs. The impact of hyperphosphorylation on RNA binding is the same as that observed for N234 (see Fig. 4 and fig. S8 for ^13^C-^1^H and ^15^N-^1^H spectra, respectively), with complete abrogation of binding in the SRPK1/GSK-3 phosphorylated form of the construct (Fig. 4, D and E), again identifying N2 and N3 as the minimum necessary elements for this inhibition. We cannot exclude the idea that N5, which we have not studied here and which contains a lysine-rich region, plays a role in RNA binding or assembly.

The possible origin of this differentiation between the impact of PKA phosphorylation and SRPK1/GSK-3 phosphorylation is revealed by comparison of ^15^N-^1^H and ^13^C-^1^H CSPs in the two cases (Fig. 4, F and G). Although much smaller shifts due to PKA phosphorylation are seen in general, the main interacting regions appear to involve the RNA binding finger (residues 90 to 106). CSPs involving the baseplate (residues 48 to 66 and 148 to 174) are considerably smaller. Notably, the region of pSR situated between positions 182 and 191 comprises four phosphorylation sites in pN234(II), but is unmodified by in vitro PKA phosphorylation (Fig. 4H). Together, these observations are consistent with a model whereby phosphorylation of the N-terminal strand of pSR (residues 182 to 191) is responsible for interaction with the baseplate, while phosphorylation of the C-terminal strand (residues 194 to 206), present in both pN234(II) and PKA-phosphorylated N234, would be responsible for binding the RNA binding finger (Fig. 4I). It is tempting to speculate that occupation of both interaction sites is required to abrogate interaction with RNA. Note that the specific differences in phosphorylation pattern of residues 194 to 206 may also contribute to stronger and more efficient binding of N2.

### Hyperphosphorylated SR sites specifically abrogate RNA binding

**Fig. 5.**
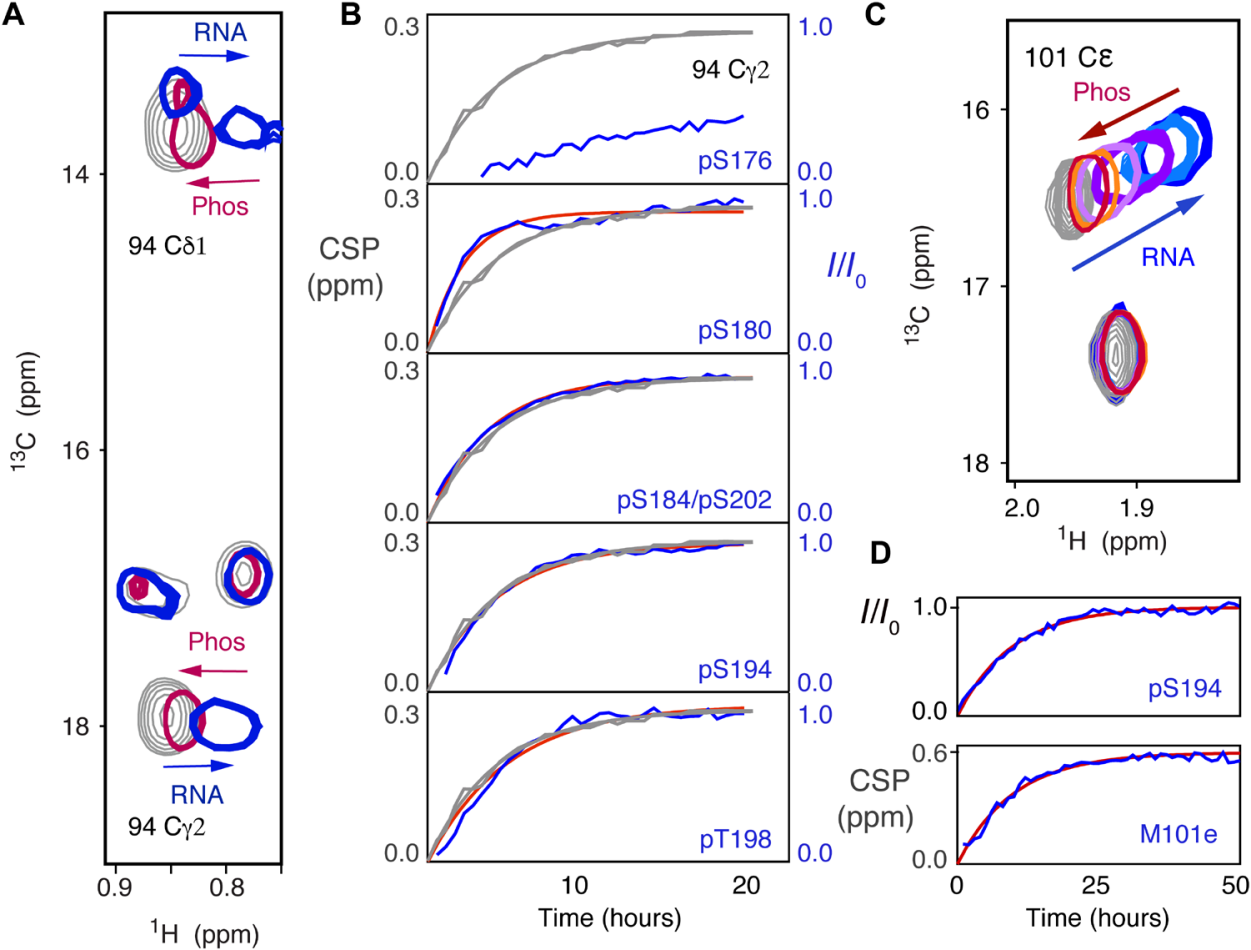
Real-time observation of RNA binding inhibition by SR phosphorylation. (**A**) CSPs in methyl ^13^C-^1^H HMQC of pN234(I), specifically 94Cγ2, showing the impact of 14-mer RNA binding (gray, 0 μM, blue 200 μM), and subsequent shift of the same peaks back toward the unbound form during incubation with GSK-3 (red). (**B**) Comparison of time course of intensity increase in the ^15^N-^1^H HSQC phosphorylation peaks from *p*SR (blue, experimental; red, single exponential fit) and the CSP of 94Cγ2 (gray, experimental and fitted). Associated time constants of (0.50 ± 0.07) hours (pS180), (0.30 ± 0.04) hours (pS184/pS202), (0.26 ± 0.07) hours (pS194), and (0.24 ± 0.05) hours (pT198) compared with (0.24 ± 0.03) hours and (0.26 ± 0.03) hours for 94Cγ2 and 94Cδ1, respectively. pS176 and pS186 (not shown) were too slow to be accurately fitted. (**C**) CSPs in methyl ^13^C-^1^H HMQC of pN234(I) (M101Cε) showing the impact of 30-mer polyA RNA binding (gray, 0 μM, blue 120 μM), and subsequent shift of the distribution of differently phosphorylated states toward the unbound, fully phosphorylated form during incubation with GSK-3 (light blue to red). (**D**) Comparison of (top) the time course of increase in intensity in the ^15^N-^1^H HSQC pS194 peak (blue, experimental; red, fitted to a single exponential) and (bottom) the shift of methyl ^13^C-^1^H CSP of 101Cε back from RNA-bound to unbound form during incubation with GSK-3 as shown in (C) (blue, experimental; red, single exponential fit). Associated time constants: (0.10 ± 0.04) hours pS194 and (0.099 ± 0.030) hours for 101Cε.

To further identify the specific phosphorylation steps that are essential for abrogating RNA binding to pN234(II), modulation of the RNA binding equilibrium was investigated during the phosphorylation process. Having established that the minimum phosphorylation level for abolishing RNA interaction occurs as a result of phosphorylation by SRPK1 and GSK-3, the 14-mer RNA was added to pN234(I), to which GSK-3 and ATP were added, to simultaneously observe the level of phosphorylation and RNA binding. Signature peaks reporting on single-stranded 14-mer RNA binding were followed using ^13^C-^1^H HMQC methyl spectra, and the level of phosphorylation was followed by measuring intensities of interleaved ^15^N-^1^H SOFAST HSQC, as described above. This allows us to follow the population shift between free and bound RNA by measuring sites that shift upon RNA binding (for example, the methyl group of I94, Fig. 5A), and compare this with the rate of appearance of the eight sites that are phosphorylated by GSK-3, where the covalent nature of the modification provides a direct measure of the extent of the phosphorylation.

The rates of phosphorylation are indicated in Fig. 5B, showing time constants of (0.50 ± 0.07) hours for pS180, (0.30 ± 0.04) hours for pS184/pS202, (0.26 ± 0.07) hours for pS194, and (0.24 ± 0.05) hours for pT198. pS176 and pS186 were too slow to be accurately fitted. Note that the level of phosphorylation of individual sites was investigated by also observing the intensity of the nonphosphorylated peak following the experiment (fig. S9), and where possible, both the increase in intensity of phosphorylated and the reduction in intensity of nonphosphorylated peaks were simultaneously fitted to the same time constant. Examples (fig. S9) indicate that with respect to experimental noise, phosphorylation is eventually complete.

Increases in intensity of resolved phosphorylation peaks are compared to the apparent chemical shift evolution of the methyl groups (Fig. 5, A and B). This comparison reveals that while S180 phosphorylates faster than the observed shift reporting on the RNA binding equilibrium, a number of sites show rates of phosphorylation that are very similar to the rate of RNA unbinding (S184, S194, T198, and S202), and two sites have slower phosphorylation rates (S176 and S186), suggesting that the latter two sites are not essential for the inhibition of RNA binding, at least under the conditions we have tested in vitro.

The experiment was repeated using 30-mer polyA RNA. In this case, we observe evolution of the chemical shift of Met 101ε, a peak that is sensitive to RNA binding, but relatively insensitive to phosphorylation, with a similar time constant compared to the intensity of the phosphorylation peak of pS194 (Fig. 5, C and D). Complete phosphorylation of pN234(I) with the eight additional sites necessary to reach pN234(II) requires intermediate creation of at least nine distinct species, comprising between zero and eight phosphorylation sites, that do not exchange with each other. The observed spectrum is therefore a superposition of differently phosphorylated states that each represents potentially different exchange regimes between free and RNA-bound protein, and as the population distribution changes from nonphosphorylated to fully phosphorylated, this spectrum evolves. We observe a distribution of peaks whose maximum shifts, from the known RNA-bound chemical shift of nonphosphorylated N234, to the chemical shift of free pN234(II), with a similar characteristic time constant as the buildup of phosphorylation. As the chemical shifts of pN234(I) and pN234(II) are very similar, the most likely explanation is that different transiently created phosphorylation states experience different exchange rates between free and RNA-bound forms, likely associated with a reduction in affinity, which gives rise to chemical shifts that are increasingly similar to the fully phosphorylated/unbound state.

Overall, simultaneous observation of the time dependence of RNA binding and SRPK1/GSK-3 phosphorylation reveals that increasing phosphorylation weakens RNA binding, until six of the eight phosphorylation sites are modified, at which point RNA can no longer bind.

### Long-range order in N is modulated by hyperphosphorylation

**Fig. 6.**
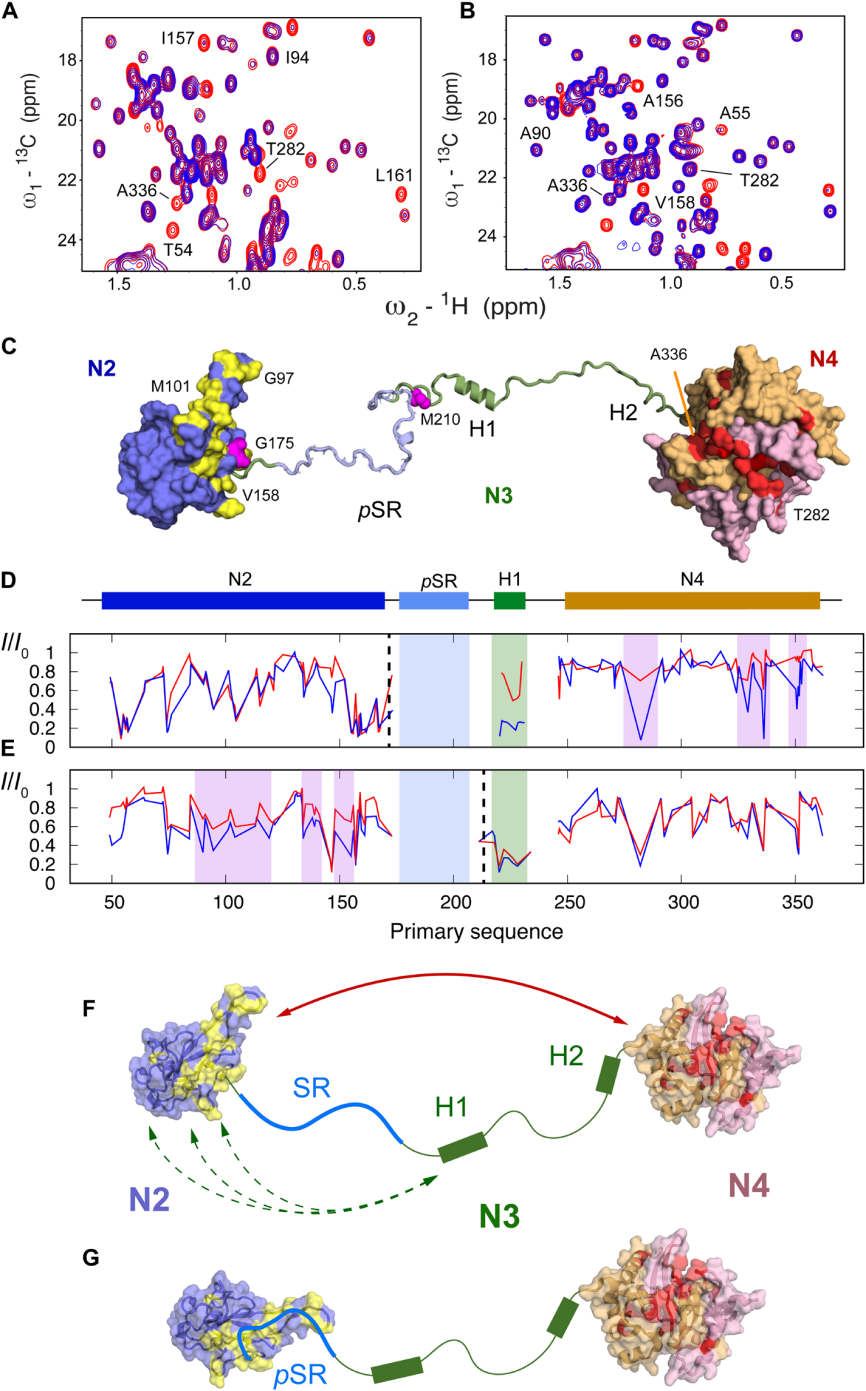
Modulation of paramagnetic relaxation enhancement due to hyperphosphorylation. (**A**) Comparison of ^13^C-^1^H HMQC in oxidized (blue) and reduced (red) forms of TEMPO-labeled N234 (G175C). (**B**) Comparison of ^13^C-^1^H HMQC in oxidized (blue) and reduced (red) forms of TEMPO-labeled pN234(II) (G175C). Strong interdomain PREs highlighted in (A) (282 and 336) are considerably weakened due to phosphorylation. (**C**) Surface representation indicating selected effects on long-range order in N234. Red: residues on hydrophobic surface of N4 dimer (the two monomers are otherwise colored pink and beige) that broaden in the presence of TEMPO label (175), an interaction that is weakened upon phosphorylation. Yellow: residues on N2 that shift upon hyperphosphorylation. Sites of the TEMPO labels are shown in magenta. (**D** and **E**) Intensity ratios between paramagnetic and diamagnetic forms of the protein. Blue, free N234; red, pN234(II). Dashed lines indicate position of the TEMPO label (175 and 210). (**F** and **G**) In the free, nonphosphorylated form (A), long-range interactions between N2 and N4 and between H1 and N2 are revealed from PREs. Upon hyperphosphorylation, interactions are weakened (B) or completely suppressed. CSPs on N2 (yellow) [see (C)].

To develop further insight into the origin of CSPs observed upon phosphorylation, and to further investigate long-range interactions within N234, we have measured paramagnetic relaxation enhancements (PREs) of cysteine mutants of N234, labeled with 4-maleimido-TEMPO. Positions for cysteine mutation were selected on N2 (175) and N3 (210) (Fig. 6).

In nonphosphorylated N234, a continuous hydrophobic surface of N4 (Fig. 6), covering residues 333 to 340, 350, and 282, is broadened by the label positioned at residue 175, indicating that this region is involved in a long-range contact between the two folded domains. In addition, the linker region, in particular the peaks corresponding to the hydrophobic groups of H1, is strongly broadened. The suggestion that H1 interacts transiently with N2 is supported by data from the paramagnetic label at position 210, which induces broadening in N2, both in the RNA binding finger (residues 90 to 106) and in the C-terminal region (residues 145 to 170) and is supported by recent similar observations using PREs from neighboring sites in a shorter construct (*30*). Broadening is also observed on the hydrophobic patch on N4 due to the label at position 210.

^15^N chemical shifts of N2 were compared in the presence and absence of N1, N3, and N4. Small differences are observed between free N2 and the longer constructs (fig. S10), clustering mainly around the baseplate residues 60 to 70 and 160 to 170, located on a single continuous face of N2, suggesting that the weak interactions with N4 observed from PREs involve this surface.

To probe the impact of phosphorylation on long range order, two forms of the TEMPO-labeled protein (positions 175 and 210) were also hyperphosphorylated [pN234(III)] and PREs were again measured (Fig. 6). Note that the high quality of the PRE data is highlighted by the highly reproducible intra-domain PRE intensity ratios (between oxidized and reduced forms) within N2, for the TEMPO label positioned at residue 175. In terms of intradomain contacts, the broadening profile of the probe on N2 reveals systematic abrogation of the long-range contacts between N2 and the hydrophobic patch on N4. This surface participates in pSR interactions with N2, suggesting that the long-range contacts between N2 and N4 are removed due to the presence of bound pSR on the surface of N2. In addition, the enhanced broadening in H1 due to the label on N2 is weakened due to hyperphosphorylation, indicating that interactions of H1 with the surface of N2 are also abolished. This is supported by abrogation of PREs due to the 210 label that implicate the RNA binding finger. The PRE of N4 due to 210 is not strongly affected by phosphorylation.

Small-angle x-ray scattering (SAXS) has also been measured on N234, pN234(III), and pN234(PKA). Although the SAXS curves show slight differences, in particular in the inverse distance ranges around 0.25 and 1.0 nm^−1^, indicating a possible redistribution of the adjacency of different domains of the protein, the overall dimensions are very similar (fig. S11). This is compatible with the observations presented above: Using PREs and CSPs, we identify contacts between the pSR region and N2 in the hyperphosphorylated protein, which would result in some compaction; however, at the same time, weak long-range contacts between N3/N4 and N2 are abrogated, resulting in larger distances between scattering centers. This combination appears to result in similar average radii of gyration, as estimated from analysis of the Guinier region of the SAXS curves. We also compare PKA-phosphorylated N234, whose scattering curve is again very similar to nonphosphorylated N234 (fig. S11).

The relevance of this abrogation of weak long-range contacts may be related to assembly or disassembly of nucleocapsids. In this respect, we note that we used negative staining electron microscopy to reveal the presence of cage-like particles upon addition of RNA (fig. S12). Such structural elements have been previously identified as substructures of the viral nucleocapsid (*15*, *16*). The presence of these assemblies was not observed when RNA was added to hyperphosphorylated N234, confirming recent observations (*47*). In the absence of atomic resolution structures of assembled nucleocapsids, it is not yet clear whether this impact on the formation of larger assemblies is related to the effect of phosphorylation on long-range contacts in N.

## DISCUSSION

The SR region of SARS-CoV-2 nucleocapsid protein has been shown to be hyperphosphorylated in infected cells, and unphosphorylated during viral assembly and when bound to genomic RNA, and nucleocapsids present in the virion are not phosphorylated (*54*, *55*). The level of phosphorylation of N is thought to act as a functional switch in the replication cycle, in genome unpackaging (following infection) or packaging (before viral assembly) (*14*, *57*–*60*). Phosphorylation was shown to affect compactness of RNP complexes (*61*), and binding of isolated N3 to long RNAs was also affected by phosphorylation (*62*). Fluorescence polarization also detected weaker binding to RNA as a result of hyperphosphorylation (*47*). A recent study proposed the sequential phosphorylation of N by host kinases SRPK1, GSK-3, and CK1 (*60*). Inhibition of SRPK1 was shown to reduce replication, while blocking the activity of GSK-3 reduced viral replication in cells and lowered infection in patients (*71*). Mass photometry and electron microscopy were recently combined to reveal that phosphorylated N forms less compact viral RNP particles (*61*).

Despite this intense interest, little is currently known about the molecular impact of hyperphosphorylation on the structural, dynamic, and functional behavior of N, and it is not yet clear where phosphorylation plays a role in the viral cycle of SARS-CoV-2. This study exploits the site-specific information provided by high-resolution NMR to describe the effects on local and long-range structure as a function of the proposed phosphorylation cycle, as well as the implications of sequential posttranslational modifications on molecular function, particularly RNA binding.

Our analysis substantiates the reported impact of phosphorylation by the previously identified putative triplet of host kinases of SRPK1, followed by GSK-3 and CK1 (*59*, *60*). This confirms that the proposed sites are indeed phosphorylated (S206 and S188 by SRPK1, followed by S176, S180, S184, S186, S190, S194, T198, and S202 by GSK-3 and S193, S197, S201, and S205 by CK1). The order of phosphorylation by GSK-3 reveals that S180 is phosphorylated first, before near-simultaneous phosphorylation of S184, S190, S194, T198, and S202, followed by slower phosphorylation of S176 and S186. These observations are confirmed by monitoring the peaks reporting on the amide group of the directly phosphorylated amino acid, as well as of the immediately neighboring amino acids (fig. S13).

Phosphorylation affects the dynamic properties of N locally, in the SR hyperphosphorylation domain. The progressive increase of rotating frame relaxation rates (Fig. 2) as a function of increasing levels of phosphorylation may result from the rigidification of the local structure in pSR, owing to the increased presence of bulky phosphate groups, and/or from interaction with N2. The latter appears to be the case, with hyperphosphorylation inducing a very similar profile of CSPs compared to those observed due to RNA binding to N234 (Fig. 3). These changes are predominantly evident not only in N2 but also in the C terminus of N3, comprising a lysine-rich helical region (H2) that is formed in the presence of N4 and that folds into a helical conformation when N3 binds to nsp3 (*49*). It therefore appears that pSR binds via the same interface on N2 as that exploited by RNA. While phosphorylation by SRPK1 has no notable impact on the N:RNA interaction, phosphorylation by GSK-3 inhibits binding completely. Time-resolved NMR, at residue-specific and atomic resolution, allows us to identify which phosphorylation sites are essential for this inhibition, including most sites modified by GSK-3, with the exception of S176 and S186. The necessary phosphorylation pattern for inhibition of RNA binding is thereby revealed as a regularly spaced charge distribution with respect to the primary sequence: S180, S184, S188, S190, S194, T198, S202, and S206, underlining the probable role of electrostatics in this inhibition. These sites are all known to be highly conserved within the SR region.

Note that while a recent single-molecule characterization of the interaction of N123 with RNA suggested that N1 may play a regulatory role (*32*) in RNA binding, atomic resolution mapping reveals that the impact of N1 and N4 on the N2 RNA binding interface is negligible, at least for the RNAs used here, with essentially identical chemical shifts and apparent affinities observed in constructs comprising N123 and N234. This comparison also confirms N2 and N3 as the minimum necessary elements for phosphorylation-dependent inhibition of RNA binding.

Progressive phosphorylation of SR therefore appears to act as a conformational and functional switch for SARS-CoV-2 nucleocapsid protein, with time-resolved NMR providing crucial insight into the role of each phosphorylation site in the functional mechanism. It is tempting to suggest that recruitment of host kinases may thereby participate in releasing genomic RNA from encapsidation, upon unpackaging from the infecting virion after cell entry. The observation that SRPK1 and GSK-3 are apparently sufficient to achieve this functional change also raises the question of the role of CK1 phosphorylation in viral function.

More insight into this putative interaction between pSR and the RNA binding interface of N2 is provided by comparison with the impact of in vitro phosphorylation by PKA. Under our experimental conditions, this results in the phosphorylation of S180, S193, S194, S197, S201, and S202, generating a possible addition of 12 negative charges in the SR region, compared to the 16 that are required to abrogate RNA binding upon SRPK1/GSK-3 phosphorylation. Crucially, however, phosphorylation by PKA has no measurable impact on RNA binding, suggesting that the pattern resulting from the physiologically relevant kinases is indeed specific for inhibition, either by binding tighter to the surface of N2, by occupying more RNA binding sites on N2, or by ensuring a uniform electrostatic screening of the RNA binding surface. A recent study provided evidence that phosphorylation of N(1 to 209) with PKA resulted in chemical shifts on the RNA binding finger (90 to 106) of N2 (*46*). Our measurements confirm this observation. Differences in the chemical shifts induced by phosphorylation with PKA and SRPK1/GSK-3 suggest that the PKA phosphorylated SR does not interact with the baseplate regions (residues 48 to 66 and 148 to 174) as is the case for SRPK1/GSK-3 phosphorylated SR. Phosphorylated SR interacts with the RNA binding finger in both cases, although inducing much smaller CSPs in the case of PKA phosphorylation. This allows us to propose distinct interaction sites between the C- and N-terminal strands of pSR with the RNA binding finger and baseplate, respectively (Fig. 4). This occupation of additional RNA binding sites on N2 by SRPK1/GSK-3 phosphorylated N234 may explain why the pSR-N2 interaction necessary to abrogate RNA binding is effective in this case and not in the case of PKA phosphorylation.

Paramagnetic relaxation NMR was used to characterize the modulation of long-range order induced by phosphorylation. In the free, unmodified protein, helix H1 interacts transiently with the surface of N2, possibly mediated by hydrophobic interactions between the leucine-rich motif at the N terminus of H1 and hydrophobic sites on N2, and specifically involving the RNA binding finger (90 to 106) and the (145 to 170) region, also implicated in RNA binding. Helix H1 has previously been implicated in binding to N2 (*30*); indeed, we find some evidence of abrogation of transient contacts with N2 as a result of hyperphosphorylation, although the negligible effect of phosphorylation on transverse relaxation of H1 and the lack of CSP in N2 in the presence or absence of N3 suggest that such interactions must be weakly populated.

Stronger interactions are also observed between N2 and N4, specifically involving a hydrophobic patch on one side of the dimeric interface of N4, comprising residues 333 to 340, 350, and 282, and the baseplate of N2, suggesting a highly dynamic equilibrium between different states, as previously proposed (*49*). This equilibrium is modified upon hyperphosphorylation, probably because the N2 interaction surface is occupied by the pSR region. Clear differences can be identified, with notable weakening, even disappearance, of the interactions implicating the hydrophobic patch on N4. This abrogation of contacts within a single N dimer may correlate with the macroscopic observation of looser nucleocapsid structures derived from electron microscopy (*61*), or the reported modulation of the dynamics of membraneless organelles that can be formed upon mixing N and RNA under certain conditions (*26*, *35*). We note that under our experimental conditions, cage-like structures are evident by negative staining electron microscopy upon mixing RNA with nonphosphorylated N234, but that such structures are absent for hyperphosphorylated N234/RNA mixtures. Nevertheless, the similarity of SAXS measured on nonphosphorylated and differently phosphorylated N234 suggests that the binding of pSR to N2 compensates for the simultaneously abrogated weak long-range contacts between the three domains and that the overall dimensions, as measured by the average radius of gyration, do not change.

Phosphorylation of disordered peptides is known to facilitate rapid switching of biomolecular function of many physiological processes (*72*–*74*). Progressive phosphorylation of newly unpackaged N by host kinases likely maintains the protein in a form that is incapable of binding RNA, via an autoinhibitory strategy that protects from nonspecific binding to cytosolic host RNA, as well as premature binding to viral genomic RNA. The clear identification of a threshold of phosphorylation, achieved during GSK-3 phosphorylation, identifies specific molecular characteristics of this switching mechanism, a process that establishes phosphorylated N in an RNA-unbound form that may participate in additional functional activity such as regulation of transcription (*14*). Newly expressed N is apparently not phosphorylated before encapsidation, assembly, and packaging, suggesting that a strategy for inhibition of phosphorylation must be available. It has been suggested that replication occurs within membraneless organelles formed by LLPS of N (*6*, *26*, *27*, *33*–*36*, *38*, *65*, *75*, *76*), and formation of organelles may provide some kind of protection from host kinases. Assuming encapsidation of newly synthesized RNA can occur within such organelles, as is the case for numerous negative strand RNA viruses (*63*, *64*, *77*–*79*), such a mechanism would provide an efficient means to escape from host kinase phosphorylation and facilitate efficient encapsidation of genomic RNA by unphosphorylated nucleocapsid protein.

In conclusion, we have used NMR spectroscopy to describe the impact of phosphorylation on the serine-arginine–rich region of the central disordered domain of SARS-CoV 2 nucleocapsid protein, a region that is hyperphosphorylated in infected cells. The proposed phosphorylation scheme, comprising SRPK1, GSK3, and CK1, is shown to abrogate binding of RNA. The phosphorylated SR domain interacts with the RNA binding domain of N via the same interface as single-stranded RNA, suggesting an auto-inhibitory mechanism involving direct competition with RNA. Long-range interactions between the folded domains of a single N protomer are also abrogated by phosphorylation, an observation potentially related to packaging or unpackaging of the viral genome. The observed impact of phosphorylation of SARS-CoV-2 N on RNA binding reports on a mechanism of inhibition of nonspecific binding of N to viral, or host RNA, while identifying a possible unpacking mechanism whereby host kinases can be recruited to extract RNA from the nucleocapsid after infection. The ability to determine the precise threshold of the highly specific phosphorylation pattern required to inhibit RNA binding provides crucial and unique insight into the role of posttranslational modification in the viral cycle and identifies potential opportunities for inhibitory strategies for this class of viruses.

## MATERIALS AND METHODS

### Protein expression and purification

All proteins (N234 47 to 364, and N123 1 to 267) were expressed in *Escherichia coli* and purified as described previously (*49*). Single-point mutations of N234 (G175C and M210C) were made in-house by site-directed mutagenesis. 14-mer RNA (UCUAAACGAACUUU) and 30-mer polyA were purchased from Integrated DNA Technologies, San Diego.

### Phosphorylation of N123 and N234

N123, N234, and G175C and M210C mutations of N234 were incubated with SRPK1 (Sigma-Aldrich), GSK-3 (Promega), CK1 (Promega), or PKA (Promega) kinases in phosphorylation buffer: 50 mM Na-phosphate (pH 6.5), 250 mM NaCl, 2 mM DTT, supplemented with 10 mM ATP, and 10 mM MgCl_2_ at 25°C. For phosphorylation cascade reaction, kinases (SRPK1, GSK-3, and CK1) were added to the sample sequentially or simultaneously.

### Nuclear magnetic resonance experiments

Unless stated otherwise, all NMR experiments were carried out in 50 mM Na-phosphate (pH 6.5), 250 mM NaCl, 2 mM DTT or 50 mM Na-phosphate (pH 6.5), 250 mM NaCl, and 1 mM TCEP at 25°C. Experiments were acquired on Bruker spectrometers with ^1^H frequencies of 600, 700, 850, and 950 MHz. Spectra were processed with NMRPipe (*80*) and visualized using NMRFAM-Sparky (*81*).

^15^N R_1ρ_ relaxation was measured at 298 K and a ^1^H frequency of 850 MHz (N234 and pN234) and 950 MHz (N123 and pN123) using a spin lock of 1.5 kHz (*82*). Relaxation delays of 1, 10, 30, 30, 70, 120, and 200 ms were used for ^15^N R_1ρ_ of N234, and 1, 10, 20, 50, 50, 90, 140, 180, and 250 ms were used for ^15^N R_1ρ_ of N123 and included repetition of one delay. Relaxation rates were fitted using in-house software and errors estimated with noise-based Monte Carlo simulation.

#### Resonance assignment

Backbone assignment of domains N1 to N4 in the nonphosphorylated forms of N123 and N234 has been recently published (*17*, *83*). Assignment of the phosphorylated forms was performed using Band-selective Excitation Short-Transient (BEST)–type triple-resonance experiments (*84*). Side-chain assignment of N2 (*25*) and N4 from SARS CoV (*41*) has been published and served as references to complete the assignment of the methyl region of ^13^C-^1^H HMQC of N234. ^13^C^α^ chemical shifts were compared to random coil values using the recently proposed random coil server for phosphorylated protein sequences (*66*). CSPs were calculated using the following expression (*85*).

$$\Delta \delta \omega =\sqrt{{\left(\Delta \delta \omega_{N15}\right)}^{2}+{\left(6.5*\Delta \delta \omega_{H1}\right)}^{2}}$$ (1)

#### PRE experiments

PRE effects were measured from the peak intensity ratios by comparing ^15^N-HSQC and HMQC 2D spectra recorded on samples labeled with TEMPO and samples reduced by the addition of 2 mM ascorbic acid. Briefly, purified cysteine mutants were reduced with 10 mM DTT at 4°C for 12 hours and dialyzed into 50 mM phosphate buffer, pH 7.0, containing 250 mM NaCl without DTT. Tenfold molar excess of 4-maleimido-TEMPO (Sigma-Aldrich) dissolved in dimethyl sulfoxide (DMSO) was added to a 100 μM stock of the reduced cysteine mutants. The reaction was incubated for 2 hours at room temperature and then left overnight at 4°C. To eliminate the excess of TEMPO, the samples were dialyzed into NMR buffer.

#### Competition experiments

SRPK1-phosphorylated N234 in phosphorylation buffer was supplemented with 200% of 14-mer or 120% of polyA(30-mer) RNA. Kinases were added directly to the NMR tube, followed by interleaved, sequential ^15^N and ^13^C correlation spectra in the presence of GSK-3 kinase recorded using BEST-TROSY (transverse relaxation-optimized spectroscopy) (*86*) and SOFAST-HMQC (selective optimized flip‐angle short‐transient heteronuclear multiple quantum coherence) (*87*). 

#### Phosphorylation analysis

BEST-TROSY (*86*) and SOFAST-HMQC (*87*) phosphorylation peak intensity time-dependent variation *I_i_*(*t*) was fitted to the following equation

$$I_{i}\left(t\right)=I_{0}\left(1-e^{-k_{i}t}\right)+I_{t=0}$$ (2)

to extract the phosphorylation rate *k_i_* for each site. Peak intensities were extracted using NMRFAM-Sparky (*81*).

### Electron microscopy

The particles used for negative-stain electron microscopy were obtained by adding 10 μM 14-mer RNA to 10 μM N234 nonphosphorylated or phosphorylated with the three kinases, in a final volume of 20 μl. Samples were incubated for 1 hour at room temperature, then applied to the clean side of carbon on mica (carbon/mica interface), and stained with 2% sodium silicotungstate (pH 7.0). Micrographs were taken with a T12 FEI microscope at 120 kV and a magnification of 30,000.

### Small angle x-ray scattering

Samples of nonphosphorylated, PKA-phosphorylated, and SRPK1-GSK3-CK1 [pN234(III)] were buffer exchanged into NMR buffer and diluted to 0.5, 1.0, and 2.0 mg/ml and measured in batch mode. SAXS measurements were recorded at the European Synchrotron Research Facility (ESRF) in Grenoble France on the BIOSAXS beamline BM29 (*88*). Data were analyzed using in-house software.
