# Supplementary material for: A specific phosphorylation-dependent conformational switch in SARS-CoV-2 nucleocapsid protein inhibits RNA binding
Source: Sci Adv. 2024 Aug 2;10(31):eaax2323. doi: 10.1126/sciadv.aax2323 (PMC11296341; doi:10.1126/sciadv.aax2323)
Supplement: Supplementary file 1 — Figs. S1 to S13 [file sciadv.aax2323_sm.pdf]

Science Advances
AAAS

Supplementary Materials for

A specific phosphorylation-dependent conformational switch in SARS-CoV-2 nucleocapsid protein inhibits RNA binding

Maiia Botova *et al.*

Corresponding author: Martin Blackledge, martin.blackledge@ibs.fr

Sci. Adv. **10**, eaax2323 (2024)
DOI: 10.1126/sciadv.aax2323

This PDF file includes:

Figs. S1 to S13

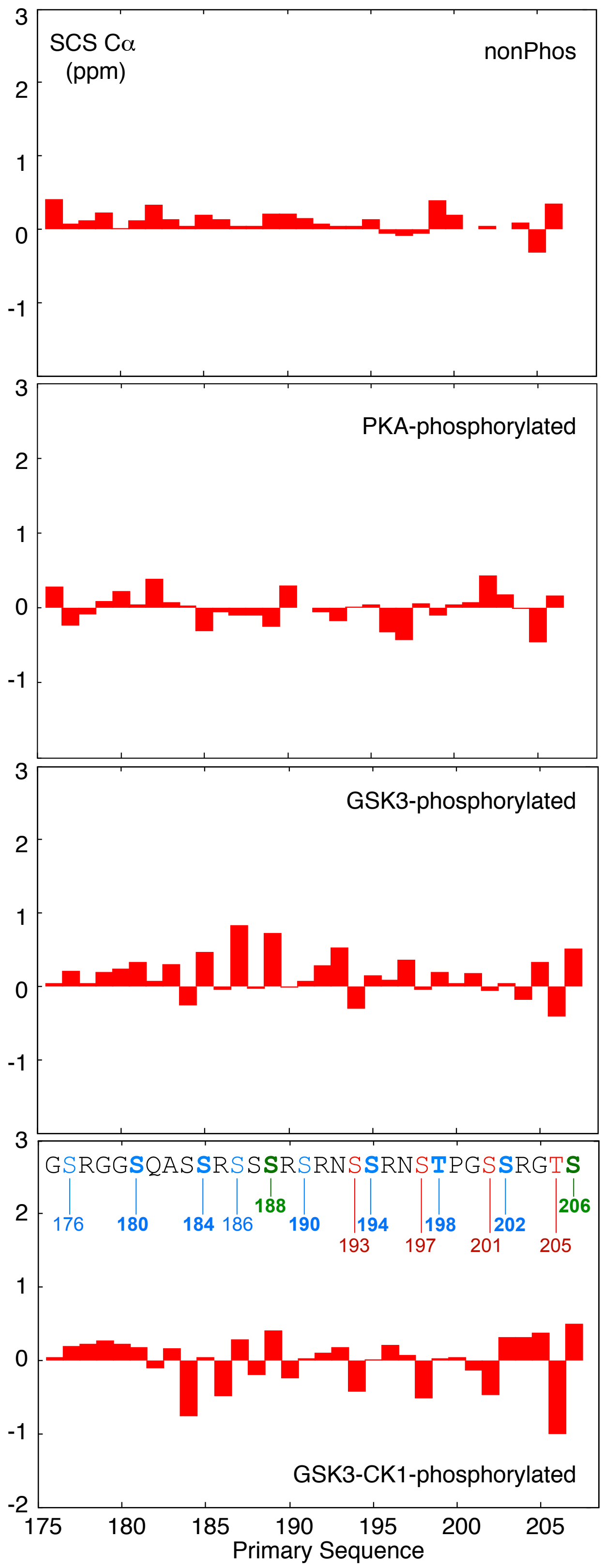

Fig. S1 **Secondary structural analysis of the pSR region.**

Secondary structural analysis of the pSR region in the non-phosphorylated form (top), PKA-phosphorylated N234, SRPK1-GSK3 phosphorylated N234 (pN234(II)) and SRPK1-GSK3-CK1 phosphorylated N234 (pN234(III), bottom). Ca shifts were compared to recently proposed random coil shifts for phosphorylated peptides (*66*). The position of phosphorylation sites due to the different kinases are shown above in green (SRPK1), blue (GSK-3) and red (CK1).

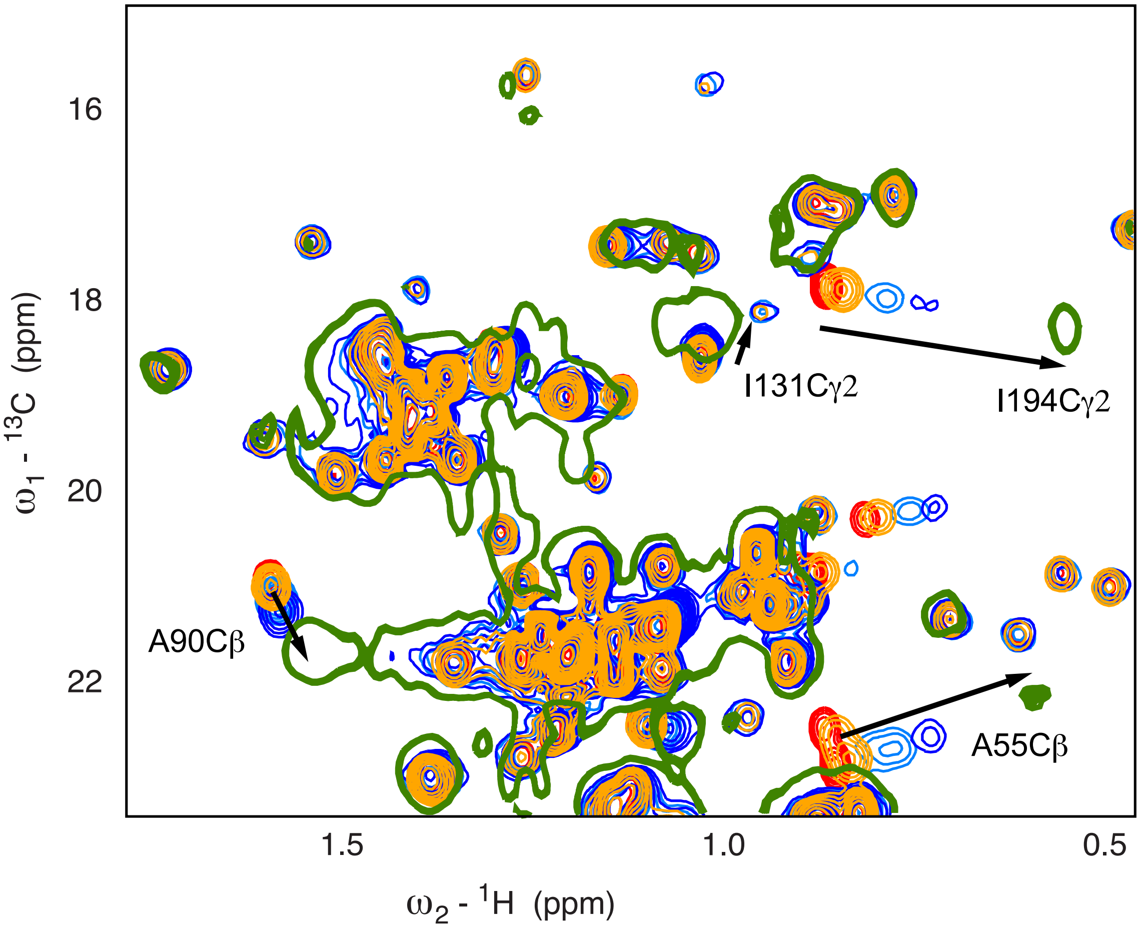

Fig. S2. **Comparison of 14mer interaction with N234 and 30mer polyA RNA.**

CSPs in ^{13}C-^{1}H HMQC N234 spectra (150µM) for selected residues as a function of 14mer single stranded RNA concentration (red – 0%, orange – 25%, light-blue 100%, dark-blue 200%) as shown in figure 4. Green chemical shift in the presence of 30mer polyA at 1:1 admixture (150µM concentration).

Numerous significantly shifted sites lie on a linear trajectory, suggesting that the bound form chemical shift is similar in both cases.

Spectra recorded at 850 MHz and 298K.

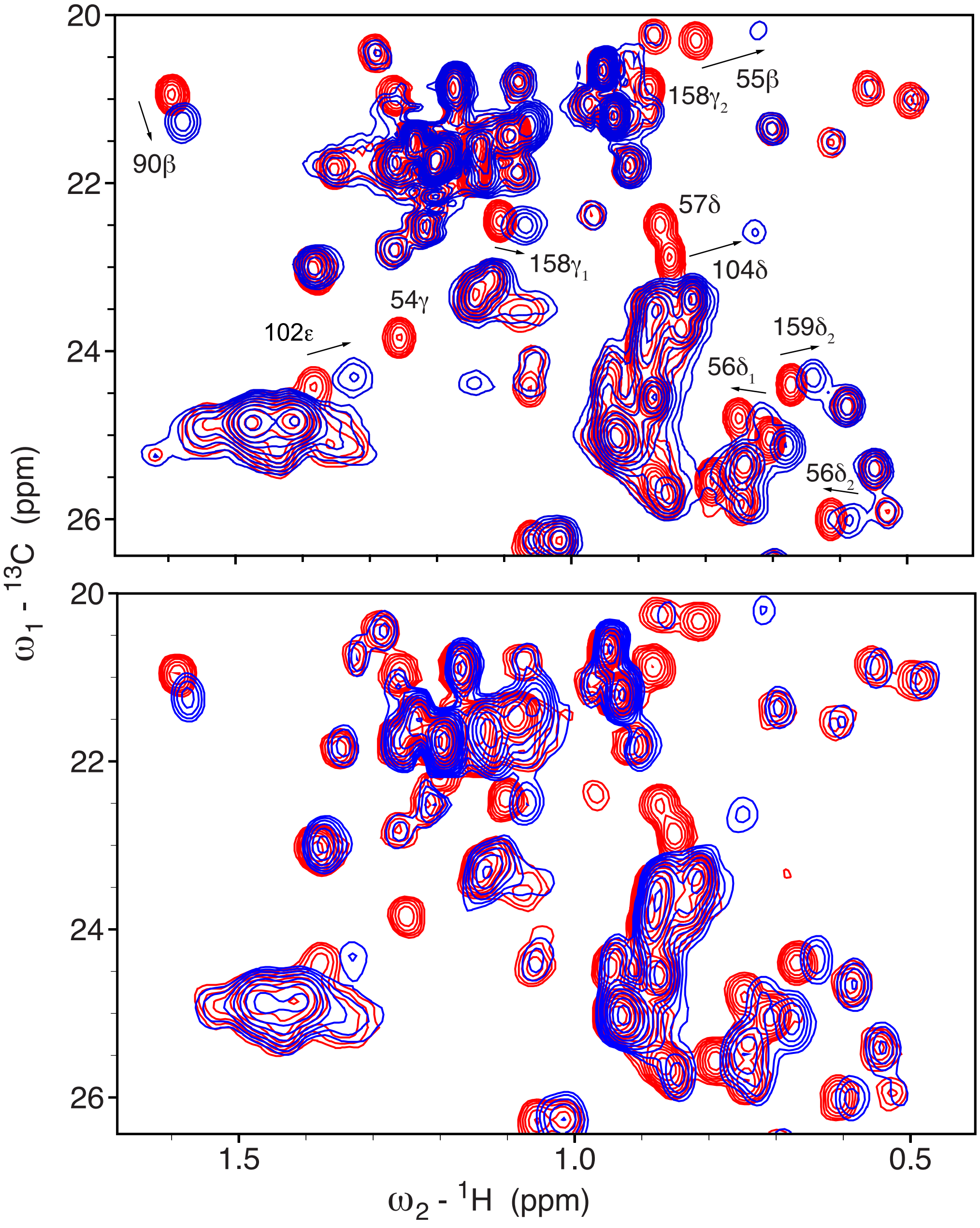

Fig. S3. **Phosphorylation by SRPK1 of N234 does not impact RNA-binding.**

Top - ^{13}C-^{1}H HMQC spectra of N234 (concentration 150 μM), free (red) and for 200% admixture of 14mer single stranded RNA (dark-blue).

Bottom - ^{13}C-^{1}H HMQC spectra of pN234(I) (phosphorylated using SRPK1, concentration 150 μM), free (red) and for 200% admixture of 14mer single stranded RNA (dark-blue).

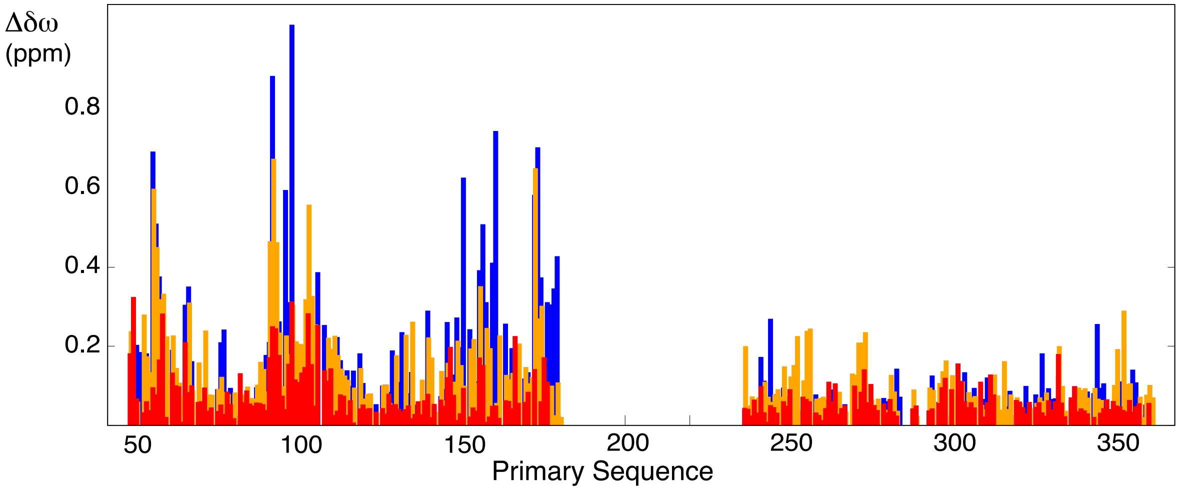

Fig. S4. **Comparison of ^{15}N-^{1}H HSQC chemical shifts due to phosphorylation by different kinases**

Comparison of ^{15}N-^{1}H HSQC chemical shifts (compared to non-phosphorylated N234) due to incubation with SRPK1 (pN234(I), red) and SRPK1/GSK-3 (pN234(II), orange) and SRPK1/GSK-3/CK1 (pN234(III), blue) and phosphorylation buffer. Phosphorylated proteins were dialysed into NMR buffer for direct comparison with the non-phosphorylated form.

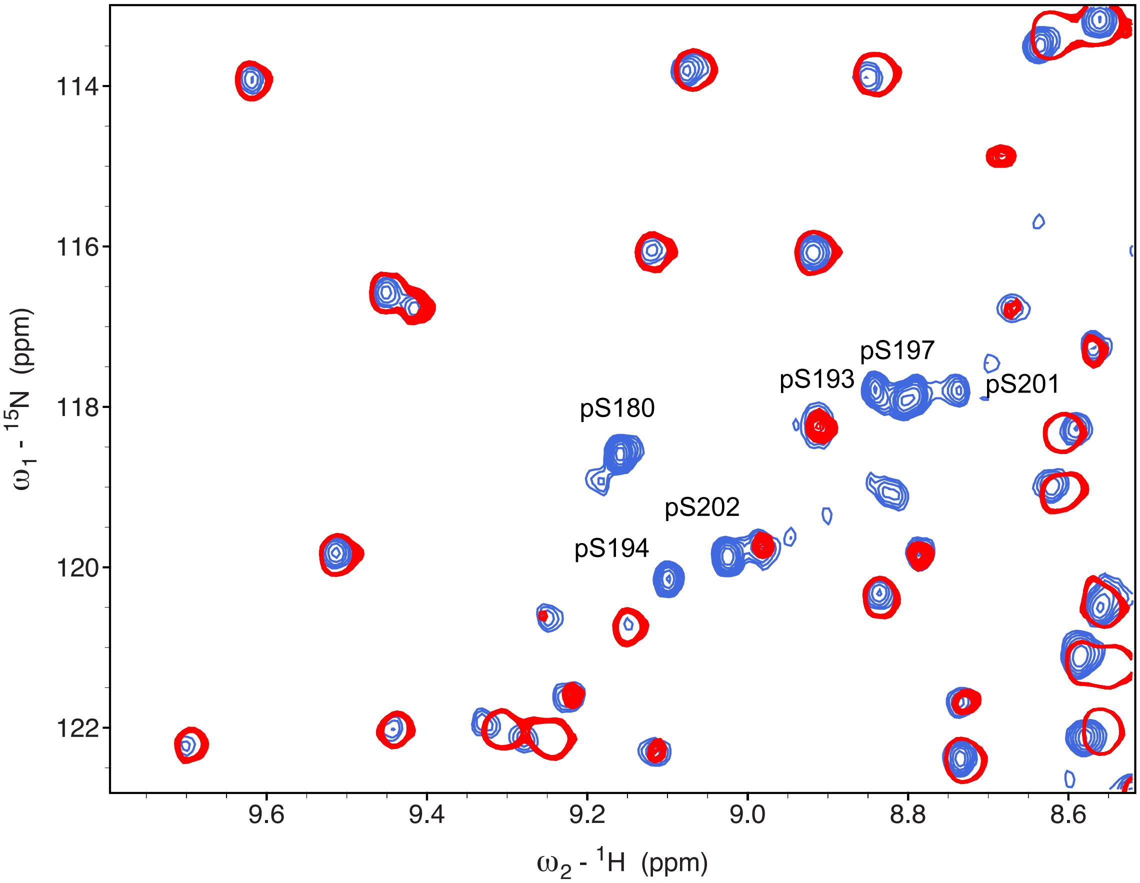

Fig. S5. **Comparison of ^{15}N-^{1}H HSQC of unphosphorylated (red) and phosphorylated (blue) N234 following incubation with PKA and phosphorylation buffer.**

Six resonances appear in the region associated with ^{15}N-^{1}H cross peaks from phosphorylated residues. These peaks were assigned using standard triple resonance methods.

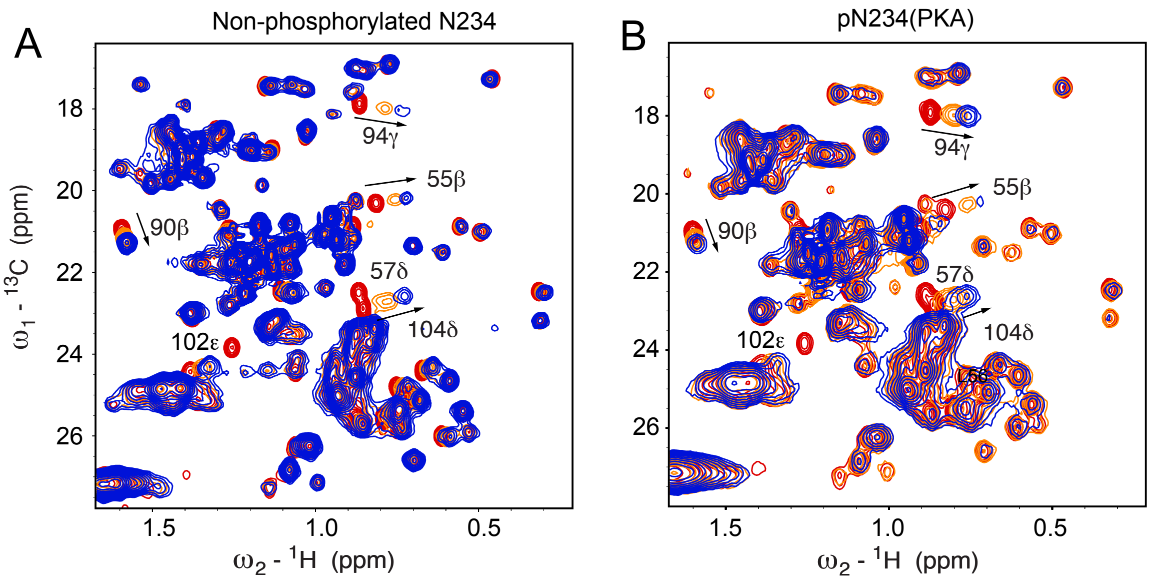

Fig. S6. **Phosphorylation by PKA of N234 does not impact RNA-binding.**

A -^{13}C-^{1}H HMQC spectra of N234 (concentration 150 μM) for increasing concentrations of 14mer single stranded RNA (red – 0%, orange – 100%, dark-blue 200%).

B - ^{13}C-^{1}H HMQC spectra of pN234(PKA) (phosphorylated using PKA, concentration 150 μM), for increasing concentrations of 14mer single stranded RNA (red – 0%, orange – 100%, dark-blue 200%).

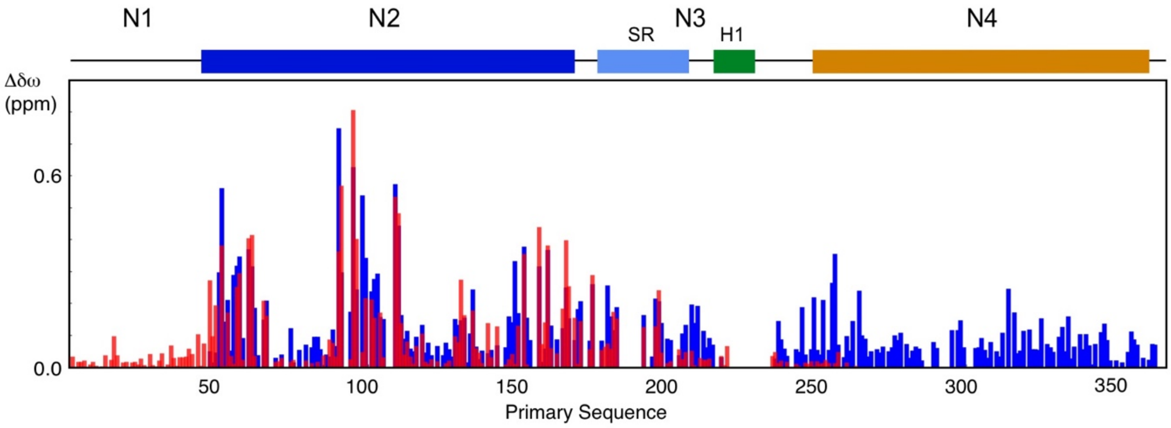

Fig. S7. **Comparison of RNA-binding to N123 and N234.**

Chemical shift perturbations (CSP) measured in the ^{15}N-^{1}H TROSY spectrum of N234 following addition of single stranded 14mer RNA (blue) in comparison with CSPs measured in the ^{15}N-^{1}H TROSY spectrum of N123 following addition of the same single stranded 14mer RNA (semi-transparent red). In the common regions (N2, N3), only residues for which CSPs were measured in both mixtures are shown. This comparison indicates that N1 and N4 do not strongly modify the RNA binding mode on N2.

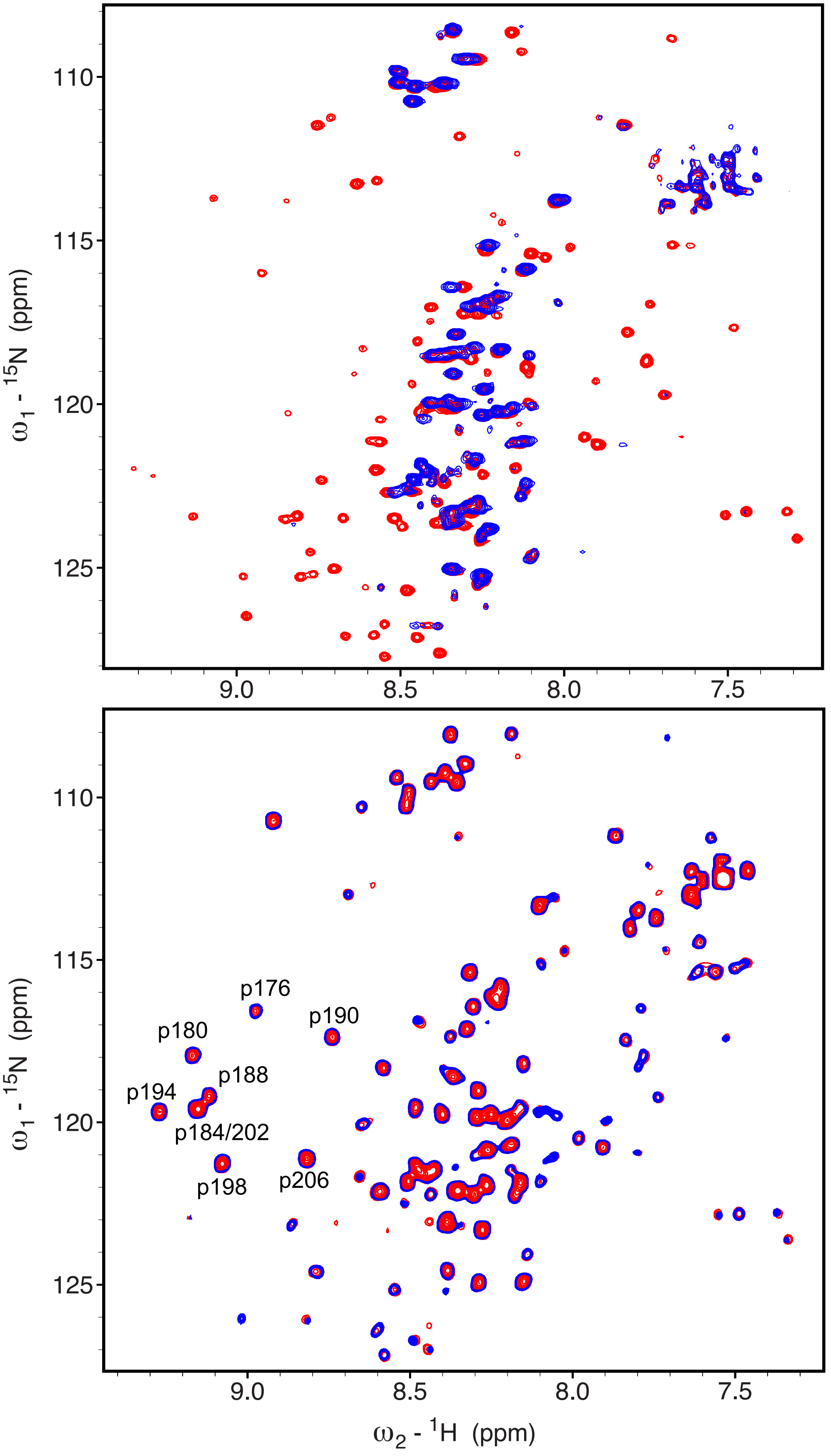

110
115
120
125
ω1 - 15N (ppm)
9.0
8.5
8.0
7.5
p176
p190
p180
p188
p194
p184/202
p198
p206
ω2 - 1H (ppm)

Fig. S8. **RNA-binding to N234 is inhibited by SRPK1-GSK3 phosphorylation.**

Top – ^{15}N-^{1}H TROSY spectra of non-phosphorylated N234 (concentration 150 μM) in the presence and absence of equimolar 14mer single stranded RNA (red – 0%, blue 200%).

Bottom - ^{15}N-^{1}H TROSY spectra of SRPK1-GSK3 phosphorylated N234 (concentration 150 μM) in the presence and absence of equimolar 14mer single stranded RNA (red – 0%, blue 200%). The 200% spectrum is presented as a single contour to highlight the lack of binding.

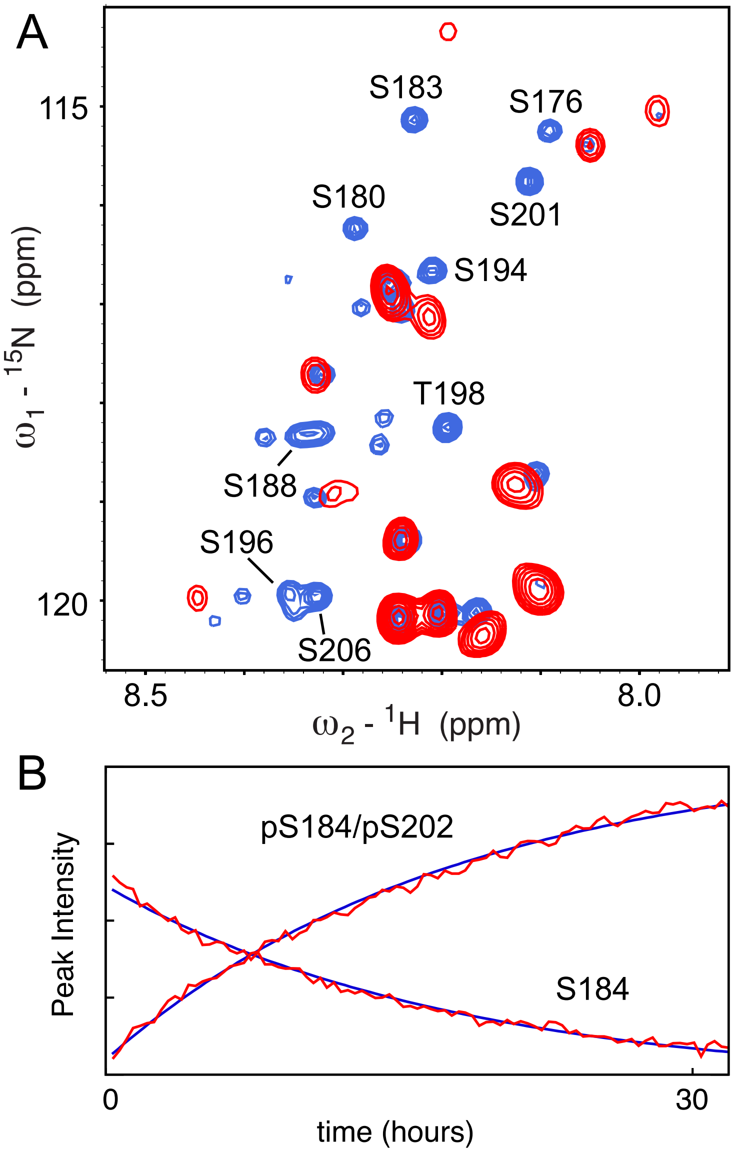

Fig. S9. **Comparison of intensities of phosphorylated and non-phosphorylated peaks.**

A – Comparison of ^{15}N-^{1}H HSQC of peak intensities for resonances corresponding to the SR region of N234 prior to (blue) and post (red) phosphorylation by SRPK1 and GSK-3 (pN234(II)), indicating that phosphorylation is essentially complete (within the signal to noise of the experiment).

B – Comparison of increase in intensity of the ^{15}N-^{1}H SOFAST HSQC of the peak corresponding to pS184/pS202 and the decrease in intensity of S202. Both curves were simultaneously fit to a single exponential describing the increase and decrease of the intensities respectively.

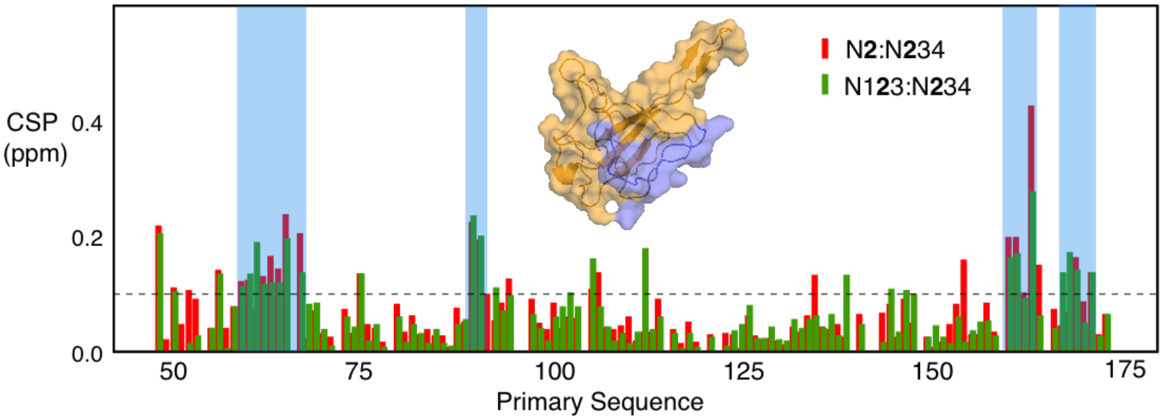

Fig. S10. **Comparison of ^{15}N-^{1}H HSQC chemical shifts of N2 in its isolated form and in constructs containing N1 and N3 and N4.**

Red - difference in chemical shifts of N2 and N2 in N234

Green - difference in chemical shifts of N2 in N234 and N2 in N123

The shading indicates regions where two out of three neighbouring residues show chemical shifts above 0.1ppm. Although small these perturbations are located on a single face of N2 (inset), suggesting that the weak interactions with N4 involve this region.

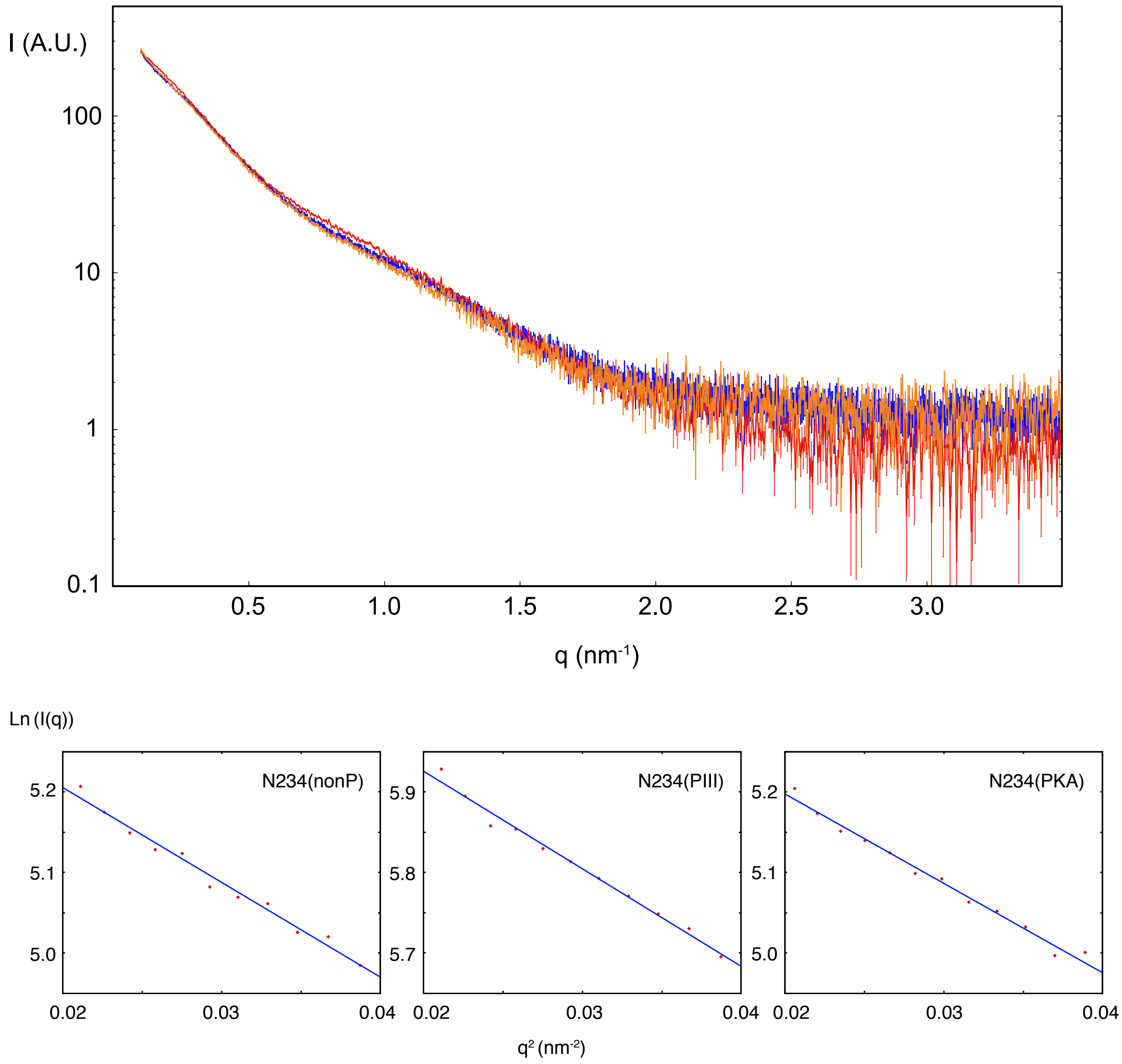

Fig. S11. **Comparison of SAXS from phosphorylated and non-phosphorylated N234**

SAXS of N234 in its unphosphorylated (blue), PKA phosphorylated (orange) and SRPK1/GSK-3/CK1 phosphorylated (pN234(III)) (red) states. The experimental curves are very similar, with slight differences in the pN234(III) state at inverse distances in the 1.0nm^{-1} range. Characteristic average radii of gyration were determined from the Guinier region of the three curves at the lowest concentration (1mg.mL^{-1}) to be very similar (PKA phosphorylated (5.84±0.12)nm, non-phosphorylated (5.91±0.14)nm and pN234(III) (5.98±0.11)nm (fits shown below).

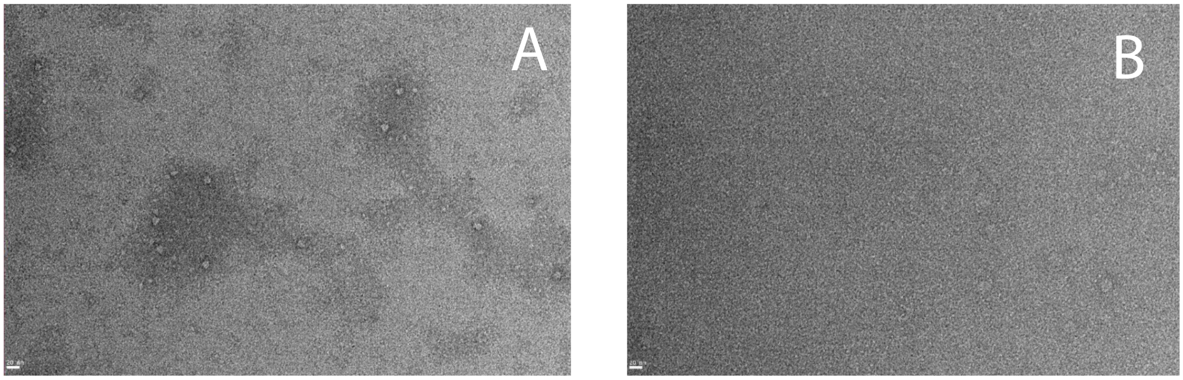

Fig S12. **Impact of phosphorylation on N234-RNA assembly**

(A) Negative staining electron microscopy of N234 in complex with 14mer RNA showing cage-like particles associated with encapsidation substructures. (B) Such particles are absent in mixtures of RNA with phosphorylated N234.

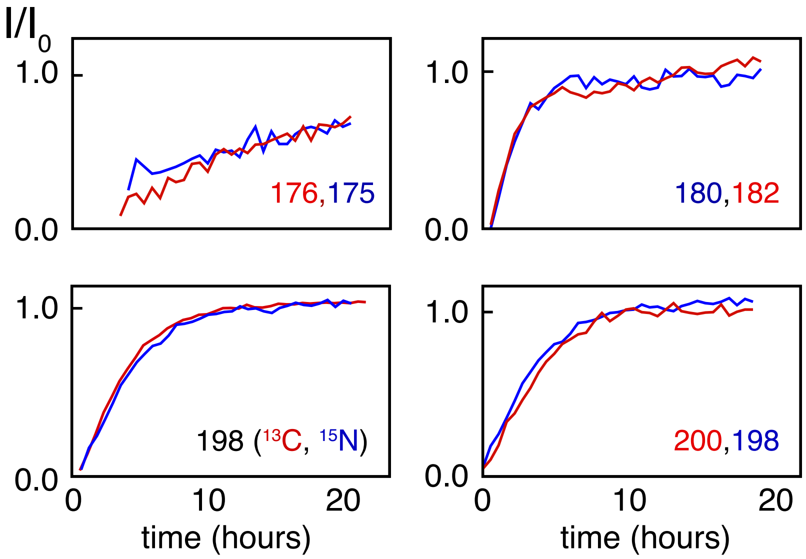

Fig. S13. **Intensity build-ups for directly phosphorylated amino acids.**

Intensity build-ups for directly phosphorylated amino acids (blue) and neighbouring or near-neighbouring residues (red) are similar. Similarly, the increase in intensity of the sidechain methyl group of T198 mirrors the behaviour of the backbone ^{15}N-^{1}H correlation peak. These comparisons provide support for the assignment and the kinetics of phosphorylation derived from the analysis of the ^{15}N-^{1}H correlation peaks of the directly phosphorylated residues.
